# Detachable dissolving microneedles loaded with *Silviavirus* phage Pelagios enhance antibacterial efficacy against methicillin resistant *Staphylococcus aureus* in *ex vivo* porcine skin

**DOI:** 10.3389/fmicb.2026.1928352

**Published:** 2026-08-21

**Authors:** Anirut Yuttagat, Arnon Songloilib, Arrita Rueangrit, Talinee Chuesanga, Lalitwadee Chotuk, Komwit Surachat, Rattanaruji Pomwised, Ekachai Dumkliang, Ampapan Naknaen

**Affiliations:** 1 Division of Biological Science, Faculty of Science, Prince of Songkla University, Hat Yai, Thailand; 2 Department of Biomedical Sciences and Biomedical Engineering, Faculty of Medicine, Prince of Songkla University, Hat Yai, Thailand; 3 Department of Ophthalmology, Faculty of Medicine, Prince of Songkla University, Hat Yai, Thailand; 4 Translational Medicine Research Center, Faculty of Medicine, Prince of Songkla University, Hat Yai, Thailand; 5 Drug Delivery System Excellence Center, Department of Pharmaceutical Technology, Faculty of Pharmaceutical Sciences, Prince of Songkla University, Hat Yai, Thailand

**Keywords:** biofilms, dissolving microneedles, phage therapy, *Staphylococcus aureus*, wound infections

## Abstract

Methicillin-resistant *Staphylococcus aureus* (MRSA) wound infections remain a major clinical challenge due to antibiotic resistance and biofilm persistence. Although phage therapy has re-emerged as a promising alternative, its efficacy is limited by poor stratum corneum penetration following conventional topical application. Here, we isolated and characterized a lytic MRSA phage, Pelagios, and evaluated its transdermal delivery using detachable dissolvable microneedles (DDMNs). Genomic analyses classified Pelagios within the genus *Silviavirus* of the family Herelleviridae and indicated that it may represent a novel species. The phage displayed rapid adsorption (∼15 min), a short latent period (∼20 min), a burst size of ∼102 PFU/cell, and stability across physiologically relevant temperature (4 °C –45 °C) and pH conditions (pH 4–10). Whole-genome sequencing confirmed the absence of toxin genes, virulence factors, antimicrobial resistance determinants, and lysogeny-associated genes, supporting its genomic safety. Pelagios exhibited broad lytic activity against multiple clinical MRSA isolates and significantly reduced planktonic bacterial populations both *in vitro* and in *ex vivo* porcine skin models in a dose-independent manner. It also effectively inhibited MRSA biofilm formation, although eradication of established biofilms was limited. Phage-loaded DDMNs fabricated from hyaluronic acid and gelatin achieved efficient skin penetration, rapid dissolution, complete needle detachment, and markedly improved phage stability at 4 °C. In *ex vivo* infection models, DDMN-mediated delivery significantly enhanced antibacterial and biofilm-eradicating efficacy compared with free phage suspension. These findings demonstrate that Pelagios delivered via DDMNs constitutes a safe and effective strategy for treating MRSA-associated wound and biofilm infections.

## 1 Introduction

The skin barrier protects the human body from invasion of exogenous pathogens, but breaches expose underlying tissue to contamination, causing infections, delayed healing, and chronic wounds linked to comorbidities such as diabetes and immunosuppression (Uberoi et al., 2024). *Staphylococcus aureus* is one of the major causative agents of wound infections worldwide and poses a major public health threat due to its extensive resistance to multiple antibiotics, particularly methicillin-resistant strains (Bessa et al., 2015; Suludere et al., 2024). Beyond antibiotic resistance, bacterial biofilms are prevalent in approximately 60% of chronic wounds, compared with only 6% of acute wounds, and thereby sustain the inflammatory phase of the healing process (James et al., 2008; Zhao et al., 2013). Both preclinical and clinical studies have reported that bacterial biofilms represent a primary cause of delayed wound healing, with *S. aureus* serving as the predominant pathogen in chronic wound infections, colonizing 93.5% of chronic leg ulcers, although most cases are polymicrobial (Zhao et al., 2013; Serra et al., 2015). Due to the high incidence of treatment failure, therapy is often supplemented with aggressive wound bed debridement to remove devitalized tissue and biofilm (Negut et al., 2018). Insufficient infection control can result in prolonged antibiotic use, extended hospitalization, amputation, sepsis, increased morbidity and mortality, and a greater risk of developing antibiotic resistance (Sen et al., 2009).

Bacteriophages or phages, viruses that specifically infect and eliminate bacteria, have emerged as a promising alternative to conventional antibiotic therapies. Phages offer several key advantages, including a high degree of host specificity that preserves commensal microbiota and the capacity for autonomous replication at the site of infection, thereby potentially enhancing therapeutic efficacy over time (Kortright et al., 2019). Staphylococcal phages, particularly those belonging to the *Silviavirus* and *Kayvirus* genera, exhibit a broad host range against clinical isolates and possess potent biofilm-disrupting capabilities, making them especially suitable for the treatment of infections such as diabetic foot infections, in which biofilms constitute a significant therapeutic challenge (O’Flaherty et al., 2005; Azevedo et al., 2022; Plumet et al., 2023; Lerdsittikul et al., 2024). Nevertheless, the application of phage therapy in wound infections remains challenging, as biofilm-associated infections can hinder effective phage penetration and limit therapeutic translation into abscess environments. Hence, improved phage delivery strategies and advanced wound-healing technologies are needed to enhance treatment efficacy.

Dissolvable microneedle technologies (MNs), comprising arrays of micron-scale needles, have emerged as promising minimally invasive transdermal delivery platforms capable of breaching the stratum corneum to facilitate the deposition of antimicrobial agents within the dermis (Li et al., 2019; Prausnitz et al., 2019). Due to their short needle length (typically 100–1000 μm), MNs enable efficient transdermal administration while minimizing patient discomfort, tissue trauma, and inflammatory responses (Jamaledin et al., 2020a,b; Shan et al., 2024). Recently, detachable dissolvable microneedles (DDMNs) have been engineered to incorporate antibacterial agents or phages for the treatment of wound infections *in vitro* and *in vivo* (Shan et al., 2024; Efendi et al., 2025). Incorporation of phages into DDMNs may therefore improve phage stability, skin penetration, and antibacterial activity against wound-associated biofilms. In the present study, we isolated and characterized a lytic MRSA phage, Pelagios, from hospital wastewater in Thailand. We evaluated its morphology, biological properties, genomic characteristics, host range, and antibacterial efficacy against planktonic and biofilm-associated MRSA. Furthermore, we developed phage-loaded detachable dissolvable microneedles to enhance transdermal phage delivery and assessed their therapeutic efficacy in *ex vivo* porcine skin infection models.

## 2 Materials and methods

### 2.1 Bacterial strains and growth conditions

A total of 17 clinical *Staphylococcus* spp. strains were used in this study, including 12 *S. aureus* strains, and 5 *S. epidermidis* strains. Clinical *S. aureus* strains were obtained from patients in the Department of Internal Medicine at Songklanagarind Hospital and Naradhiwas Rajanagarindra Hospital, Thailand. Among these, six isolates were obtained from Naradhiwas Rajanagarindra Hospital, including MRSA 40108 (tissue), 4903667 and 40149 (urine; midstream urine, MSU), 50017 (pus), 30002 (tissue), and 535896 (sputum) (Table 1). The remaining isolates were obtained from Songklanagarind Hospital, including *S. aureus* ATCC 29312 (reference strain), A7-1, A7-2, A21-1, and A21-2 (corneal ulcer isolates); *S. epidermidis* A2-1, A2-2, and A2-3 (corneal ulcer isolates); and *S. aureus* A26-1 and A26-2 (corneal ulcer isolates). All bacterial strains were stored at -80 °C. Prior to use, bacteria were cultured on Tryptic Soy Agar (TSA) plates and incubated at 37 °C. For preparation of log-phase cultures, a single bacterial colony was inoculated into Tryptic Soy Broth (TSB) and incubated overnight at 37 °C. The overnight culture was then diluted 1:50 in fresh TSB and incubated at 37 °C with shaking at 250 rpm for ∼ 3 h to obtain log-phase bacterial cells.

**TABLE 1:** The host range spectrum and efficiency of plating (EOP) of phage Pelagios against *Staphylococcus* clinical isolates.

*Staphylococcus spp.*	Strain code	Source	Phage sensitivity	EOP value
*S. aureus* MRSA	40108	Tissue	+	1.00 (high)
*S. aureus* MRSA	40149	Urine (MSU)	+	1.10 (high)
*S. aureus* MRSA	50017	Pus	+	<0.001
*S. aureus* MRSA	30002	Tissue	+	<0.001
*S. aureus* MRSA	4903667	Urine (MSU)	−	ND
*S. aureus*	535896	Sputum	+	<0.001
*S. aureus*	29312	ATCC	+	2.07 (high)
*S. aureus*	A7-1	Corneal ulcer	+	0.74 (high)
*S. aureus*	A7-2	Corneal ulcer	+	0.67 (high)
*S. aureus*	A21-1	Corneal ulcer	−	ND
*S. aureus*	A21-2	Corneal ulcer	−	ND
*S. epidermidis*	A2-1	Corneal ulcer	−	ND
*S. epidermidis*	A2-2	Corneal ulcer	−	ND
*S. epidermidis*	A2-3	Corneal ulcer	−	ND
*S. epidermidis*	A22-1	Corneal ulcer	−	ND
*S. epidermidis*	A22-2	Corneal ulcer	−	ND
*S. aureus*	A26-1	Corneal ulcer	−	ND
*S. aureus*	A26-2	Corneal ulcer	−	ND

The results “+” indicates phage-sensitivity; “−” indicates phage-resistant; ND, not determine. Displayed EOP values, phage infectivity was categorized as high (EOP ≥ 0.5), moderate (0.1 < EOP > 0.5), low (EOP ≤ 0.1), or inefficient (EOP < 0.001), respectively (Table 1).

### 2.2 Phage isolation, purification and propagation

To isolate phages specific to MRSA, influent wastewater collected from Songklanagarind Hospital was enriched using MRSA strain 40108 as the parental host, as previously described (Naknaen et al., 2026). Briefly, 20 mL of wastewater samples were first filtered through a 0.45-μm membrane and mixed with 0.6 g of TSB powder and 200 μl of a log-phase culture of the 40108 host strain was added to the filtrate. The mixture was incubated at 37 °C for 24 h with shaking at 150 rpm. Following incubation, the culture was centrifuged at 5,478 × *g* for 5 min at 4 °C, and the resulting supernatant was collected and filtered through a 0.45-μm membrane. The filtrates were then investigated for the presence of phages using the double-layer agar (DLA) method. For the DLA assay, the filtrate and host bacteria were mixed with 0.35% TSA soft agar, overlaid onto solid TSA plates, and incubated at 37 °C for 24 h. Single plaques were selected and purified through at least three times. The final purified phage lysates were resuspended in SM buffer (200 mM NaCl, 10 mM MgSO_4_⋅7H_2_O, 50 mM Tris-HCl, pH 7.5) and stored at 4 °C.

For phage propagation using the broth method, 50 μL of phage stock (1 × 10^6^ PFU/mL) was mixed with 100 μL of host bacterial culture (1 × 10^8^ CFU/mL) and added to 800 μL of TSB. The mixture was incubated at 37 °C for 24 h with shaking at 150 rpm. Following incubation, the culture was centrifuged at 11,180 × *g* for 3 min, and the resulting supernatant was filtered through a 0.45-μm membrane filter to obtain a phage lysate. The sterility of the filtrate was confirmed by spot-testing on bacterial host lawns to ensure the absence of viable bacterial contaminants. A 0.45-μm filter was used to preserve phage recovery efficiency while removing bacterial cells and debris. The filtrate was stored at 4 °C until use. For phage propagation by the DLA method, 200 μL of phage stock was mixed with 100 μL of bacterial culture and combined with 5 mL of TSB containing 0.35% (w/v) agar. The mixture was then poured onto a TSA plate and incubated at 37 °C for 24 h. After incubation, 5 mL of SM buffer was added to the web lysis and allowed to stand for 4–5 h to facilitate phage diffusion from the soft agar. The supernatant was subsequently collected, centrifuged at 5,000 × *g* for 30 min, and filtered through a 0.45 μm membrane filter to obtain purified phage lysate. The filtrate was stored at 4 °C until use.

### 2.3 Host range determination and efficiency of plating (EOP)

To determine the infectivity of the phage against clinical *S. aureus* isolates, the host range of the phage was evaluated using a spot test against 18 Staphylococcus spp. strains. Briefly, 10 μL of phage suspension (approximately 10^8^ PFU/mL) was spotted onto the surface of bacterial lawns. After incubation at 37 °C for 24 h, the formation of clear lysis zones was recorded as positive lytic activity (phage susceptible), whereas the absence of clearing indicated non-susceptibility. Clinical strains that exhibited lytic activity in the spot test were further evaluated for efficiency of plating (EOP) using DLA assay. Briefly, 10-fold serial dilutions of phage suspensions were spotted onto susceptible bacterial lawns, and plaques were observed after incubation at 37 °C for 24 h. The EOP was calculated as the ratio of plaque-forming units (PFU) obtained on each tested bacterial strain relative to those obtained on the parental host strain. Based on EOP values, phage infectivity was categorized as high (EOP ≥ 0.5), moderate (0.1 ≤ EOP < 0.5), low (0.001 ≤ EOP < 0.1), or inefficient (EOP < 0.001) (Naknaen et al., 2024). All experiments were performed in independent triplicate.

### 2.4 Phage morphological characterization by transmission electron microscopy

To observe the morphological features of the phage, transmission electron microscopy (TEM) was performed using a negative-staining method. Briefly, 10 μL of phage stock was spotted onto a bacterial lawn of the parental host and incubated at 37 °C for 24 h. The resulting clear lysis zone was excised and suspended in SM buffer, followed by incubation at 4 °C for 24 h to allow diffusion of phage particles from the agar matrix. The suspension was subsequently centrifuged at 11,180 × *g* for 3 min at 4 °C, and the supernatant was filtered through a 0.45-μm pore-size membrane filter. The sterility of the filtrate was confirmed by spot-testing on bacterial host lawns to ensure the absence of viable bacterial contaminants. A drop of the filtrate was placed onto carbon-coated copper grids and allowed to adsorb for 2 min. The grids were then negatively stained with 2% uranyl acetate for 2 min. Phage morphology was examined using a Talos F200i transmission electron microscope (Thermo Fisher Scientific, Waltham, MA, USA) operating at 200 kV.

### 2.5 Phage adsorption and one-step growth kinetics analysis

To determine the adsorption efficiency of the phage to the host surface, 900 μl of log-phase bacterial cells (1 × 10^8^ CFU/mL) were infected with 100 μl of phage lysate (1 × 10^7^ PFU/mL) in 9 mL of TSB medium at a MOI of 0.01 and incubated at 37 °C. Samples were collected at 0, 3, 6, 9, 12, 15, 20, and 25 min, immediately filtered through a 0.45 μm membrane filter to remove bacterial cells, and the titers of unadsorbed phages were determined using the double-layer agar (DLA) assay. The latent period and burst size of the phage were evaluated using a one-step growth kinetics assay, following the method described by Kropinski (2018). Log-phase host bacteria (900 μl; 1 × 10^8^ CFU/mL) were infected with 100 μl of phage lysate (1 × 10^7^ PFU/mL) at an MOI of 0.01. The mixture was incubated at 37 °C for 10 min to allow phage adsorption, followed by centrifugation at 11,180 × *g* for 3 min to remove unadsorbed phages. The pellet was resuspended in 10 mL of fresh TSB medium and incubated at 37 °C. Phage titers were determined immediately after resuspension and at 10-min intervals up to 140 min using the DLA assay. The latent period was defined as the time from the start of the experiment until the onset of phage progeny production. The burst size was calculated by subtracting the number of unadsorbed phages from the average maximum phage yield and dividing the result by the number of infected cells (PFU/CFU) (Kropinski, 2018). All experiments were performed independently in triplicate.

### 2.6 Thermal and pH stability assays

The effects of temperature on phage stability were evaluated at 4, 25, 37, 45, 55, 60, 70, and 80°C. Briefly, phage suspensions (∼10^8^ PFU/mL) prepared in SM buffer (pH 7.4) were incubated at the indicated temperatures for 2 h. Following incubation, samples were immediately cooled on ice, and phage titers were determined using the DLA assay. The relative phage titer was calculated as the ratio of phage titers obtained after each temperature treatment to those maintained 4°C. To assess pH stability, phage suspensions (∼10^8^ PFU/mL) were incubated in SM buffer adjusted to different pH values (pH 1, 2, 4, 6, 8, 10, 12, and 14) at 37 °C for 2 h. After treatment, phage samples were serially diluted, and titers were determined using the DLA assay. The relative phage titer was calculated as the ratio of phage titers at each pH condition to those in SM buffer. All experiments were performed in independent quadruplicate.

### 2.7 Phage genome analysis

The phage lysate (20 mL) was precipitated by adding 10 mL of 50% (w/v) polyethylene glycol-8000 and 10 mL of 3 M NaCl, followed by gentle mixing. The mixer was stored at 4 °C overnight. Then, the mixture was centrifuged at 5,478 × *g* for 45 min, followed by discarding the supernatant and leaving the pellet to dry. The pellet was resuspended with 400 μl of deionized water and treated with DNase I (5 units) and RNase I (1 unit) to degrade bacterial genomic DNA and RNA, followed by incubation at 37 °C for 1 h. The DNase I activity was inhibited by 5 mM EDTA at final concentration. The phage capsid was digested by 100 μl of lysis buffer (10 mg/mL proteinase K and 10% SDS) at 60 °C for 1 h. After that, phage DNA was extracted using phenol: chloroform: isoamyl alcohol (25:24:1) extraction. Briefly, an equal volume of phenol: chloroform: isoamyl alcohol was added, mixed, and centrifuged at 11,180 × *g* for 10 min. The aqueous layer was transferred and mixed with an equal volume of chloroform and centrifuged at 11,180 × *g* for 10 min. The aqueous layer was collected, mixed with 0.3 vol of 3 M sodium acetate and 1 vol of isopropanol, and stored at -20°C overnight. The mixture was centrifuged at 11,180 × *g* for 10 min, and the DNA pellet was washed with 70% ethyl alcohol, followed by discarding the supernatant, and the pellet was air-dried, dissolved with DI, and stored at 4 °C. The DNA concentration was quantified using a NanoDrop spectrophotometer, and the quality of the DNA was assessed by gel electrophoresis. DNA quality was evaluated by measuring the absorbance A260/280 ratio, and DNA concentration was measured by a NanoDrop spectrophotometer. Whole-genome sequencing was performed using the Illumina platform 150 bp. Read quality was assessed using FastQC and filtered with Trimmomatic (version 0.39) using default parameters. The reads were then assembled into contigs using SPAdes. Open reading frames (ORFs) and lysogeny-associated genes were identified and analyzed using the Galaxy server^1^ and PHASTER^2^ and subsequently annotated by BLASTp searches against the NCBI database. Intergenomic similarities between Pelagios and related phages were assessed using VIRIDIC^3^. A comparative analysis of multiple genomic loci was performed using the DiGAlign online tool^4^. Phylogenetic trees were generated based on the Genome-BLAST Distance Phylogeny (GBDP) method via the VICTOR web server^5^ and were visualized using the Interactive Tree of Life (iTOL) online tool^6^. Further prediction of the Pelagios genome antimicrobial resistance genes using ResFinder version 4.7.2^7^, toxin proteins using CSM-toxin^8^, ToxinPred3.0^9^, DBETH, and virulence factors using VFanalyzer^10^ and VirulenceFinder 2.0 web server^11^.

### 2.8 *In vitro* evaluation of the antibacterial and antibiofilm activities of phage treatments, with *ex vivo* assessment of antibacterial efficacy

#### 2.8.1 Bactericidal activity at different MOIs for 24 and 48 h

The effect of phage dosage on antibacterial activity was evaluated at multiplicities of infection (MOIs) of 0.01, 0.1, 1, and 10. One hundred microliters of bacterial suspension in TSB medium were added to each well of a 96-well polystyrene plate, followed by the addition of phage at the corresponding concentrations. Wells containing TSB alone or bacteria alone served as controls. The plates were then incubated at 37 °C, and viable bacterial counts were determined after 24 and 48 h using serial dilution and plating on agar. All experiments were performed in quadruplicate.

#### 2.8.2 Biofilm inhibition assay

The biofilm assay was performed using a modified method (Alves et al., 2014). Biofilm formation was assessed in 96-well polystyrene plates to promote cell attachment. TSB supplemented with 2.5% glucose and 1% NaCl (1% TSBg-NaCl) was used as the growth medium, as these additives enhance biofilm formation (Alves et al., 2014; Lerdsittikul et al., 2024). An overnight culture of MRSA 40108 was diluted to 1 × 10^8^ CFU/mL, and phage Pelagios was added to achieve final concentrations of 10^6^, 10^7^, or 10^8^ PFU/mL. The microplates were then incubated at 37 °C for 48 h under static conditions. After incubation, the medium was removed and the wells were gently washed twice with sterile 0.85% saline to eliminate planktonic cells. Biofilm biomass was subsequently quantified by staining with 0.1% crystal violet, and absorbance was measured at 570 nm using a microplate spectrophotometer (SpectraMax iD5 Multi-Mode Microplate Reader, Molecular Devices, USA). All experiments were performed with 24 replicates.

#### 2.8.3 Biofilm eradication assay

To establish biofilms, MRSA 40108 was grown in 1% TSBg-NaCl medium and incubated in 96-well polystyrene tissue culture microplates at 37 °C for 48 h. After incubation, the established biofilms were rinsed twice with sterile 0.85% saline solution. Phage Pelagios solutions at final concentrations of 10^7^, 10^8^, or 10^9^ PFU/mL were added to the wells and incubated at 37 °C for an additional 24 h. Biofilm biomass was subsequently quantified by staining with 0.1% crystal violet, and absorbance was measured at 570 nm using a microplate spectrophotometer (SpectraMax iD5 Multi-Mode Microplate Reader, Molecular Devices, USA). All experiments were performed with 24 replicates.

#### 2.8.4 Antibacterial effect of phage Pelagios in a wound-infection model using *ex vivo*

To evaluate the antibacterial activity of phage Pelagios on a biotic surface, the *ex vivo* pig skin model was performed with modifications (Luo et al., 2024). To establish the *ex vivo* porcine skin infection model, porcine skin was sterilized by soaking in 75% ethanol for 30 min and then cut into ∼1.5 × 1.5 cm explants. To simulate a wound, a 50 mm diameter and 1 mm deep wound bed was created in selected explants using a scalpel. To ensure a sterile environment for the duration of the assay, the explants underwent a secondary sterilization step consisting of a 1-h immersion in 75% ethanol followed by 1-h UV light exposure. The explants were placed in 24-well plates containing 1 mL of physiological saline agar [0.9% (w/vol) NaCl, 0.5% (w/vol) agar, pH 5.5]. Fifty microliters of bacterial suspension (1 × 10^8^ CFU/mL) were inoculated into each wound area, and the plates were incubated at 37 °C for 2 h to allow establishment of infection. Fifty microliters of phage solution at final concentrations of 10^6^, 10^7^, or 10^8^ PFU/mL were then applied to the infected wounds. After 24 h of incubation at 37 °C, bacteria were harvested by adding 1 mL of sterile 0.85% saline to the wound area. Viable bacterial counts were determined by plating on mannitol salt agar (MSA). All experiments were performed in quadruplicate.

### 2.9 Fabrication of phage-loaded detachable dissolvable microneedles (PDDMNs)

Phage-loaded detachable dissolvable microneedles (PDDMNs) were fabricated using a mold-casting method adapted from procedure previously described by Efendi et al. (2025), with modifications (Efendi et al., 2025). A binary polymer mixture was prepared as the microneedle matrix, consisting of 17% (w/w) hyaluronic acid (HA; molecular weight 100 kDa) and 3% (w/w) gelatin. The polymer mixture was sterilized by autoclaving at 121 °C for 15 min and subsequently cooled at 4 °C prior to use. All fabrication procedures were performed under sterile conditions. For the preparation of PDDMNs, a phage suspension was incorporated into the cooled polymer mixture to obtain a final phage concentration of ∼1 × 10^8^ PFU/mL The resulting mixture was gently homogenized to ensure uniform phage distribution throughout the polymer matrix. Subsequently, 200 μL of the phage-loaded polymer mixture was carefully cast into the needle layer. The filled mold was centrifuged at 2,165 × *g* at 4 °C for 50 min to facilitate complete filling of the microneedle cavities. After 30 min of centrifugation, an additional polymer mixture containing HA and gelatin without a phage was added onto the partially formed microneedle layer to serve as the backing layer. Following centrifugation, the molds were removed from the 3D-printed holders and transferred into a plate containing silica gel. The plate was subsequently placed in a sealed silica gel desiccation box and stored at 4 °C for approximately 3–4 days. The microneedle patches were carefully demolded and stored in airtight containers at 4 °C until further use. For the fabrication of blank dissolving microneedles, the same procedure was followed except that no phage suspension was added to the polymer mixture.

### 2.10 Physical characterization of DDMN and *ex vivo* evaluation of skin penetration and diffusion

The morphology of the microneedles was examined using a Dino-Lite digital microscope and a scanning electron microscope (SU3900, Hitachi, Japan). The penetration and diffusion behavior of the DDMNs were evaluated using cross-sectioned porcine skin treated with Rhodamine B-loaded DDMNs, following a previously described procedure (Phoka et al., 2023). Fresh porcine skin was cleaned, shaved with a hair clipper, and gently dried with tissue paper. The DDMN patch was applied to the skin and manually pressed for 10 s. Two drops of water were then placed on the backing sheet and allowed to stand for 2 min to facilitate dissolution. The patch was subsequently removed, and the treated skin was immediately sectioned along the line of microneedle insertion. Cross-sections were examined under a stereomicroscope (Olympus JP22, Japan). Complete microneedle delivery was confirmed by inspecting the patch for residual needles after removal and examining skin cross-sections at each row of red-stained sites to determine the number of embedded needles. Penetration depth was quantified by measuring the distance between the stratum corneum and the distal end of the red-stained regions using a stereomicroscope. All measurements were performed immediately after patch application to minimize dye diffusion. Each experiment was conducted in quintuplicate using five independent skin samples. For diffusion studies, red dye-loaded DDMNs were applied to porcine skin following the same procedure. After application, the treated skin was placed on moistened tissue (pre-soaked with PBS buffer) inside a covered Petri dish to prevent dehydration. At predetermined time intervals (0, 15, 30, 60, and 120 min), the skin samples were sectioned along the microneedle insertion sites and immediately examined under a stereomicroscope. Diffusion experiments were performed in triplicate using three independent samples.

### 2.11 Stability of phage Pelagios in DDMNs

To determine phage Pelagios’s stability in DDMNs, each of the freshly prepared DDMNs co-loaded with phage Pelagios were placed in 6-well plates and wrapped with aluminum foil to protect them from light. The samples were then stored at 4 °C and 25°C to evaluate phage Pelagios’s stability during storage. The samples were collected on 0, 5, 10, 20 and 30 days after storage. At each time point, the PDDMNs patches were dissolved in sterile SM buffer. The obtained phage suspensions were immediately analyzed for phage viability using the DLA assay. Phage titers were expressed as plaque-forming units per milliliter (PFU/mL). All experiments were performed in triplicate.

### 2.12 *Ex vivo* porcine skin wound infection model for evaluating PDDMN-mediated treatment against planktonic and biofilm MRSA states

To assess the antibacterial efficacy of phage Pelagios-loaded DDMNs, the *ex vivo* porcine skin wound infection model was employed as described above. Following sterilization of the skin explants, 10 μL of bacterial culture was inoculated into each wound area. The explants were placed in 6-well plates containing 2 mL of physiological saline agar and incubated at 37 °C for 2 h to allow establishment of infection. The experiment was conducted in four groups: SM buffer (control), free phage suspension (1 × 10^8^ PFU/mL), SM-loaded DDMNs, and phage-loaded DDMNs, which were applied to the infected wounds. The plates were incubated at 37 °C for 24 h. Bacteria were subsequently harvested by adding 5 mL of 0.85% normal saline solution to the wound area followed by vortex. Treatment outcomes were evaluated by determining viable bacterial counts on MSA. All experiments were performed in biological replicates.

To assess the ability of phage Pelagios-loaded DDMNs to eradicate biofilm MRSA, the *ex vivo* porcine skin model was prepared as described above. Briefly, sterile porcine skin explants were inoculated with 10 μL of MRSA 40108 suspension (1 × 10^7^ CFU/mL) and incubated at 37 °C for 48 h to allow biofilm formation. Subsequently, SM buffer (control), free phage suspension (1 × 10^8^ PFU/mL), SM-loaded DDMNs, or phage-loaded DDMNs were applied to the infected skin and incubated at 37 °C for 24 h. Biofilm biomass was then quantified by staining the skin with 0.1% crystal violet for 20 min at room temperature. Unbound dye was removed by washing with 0.85% normal saline solution, and the absorbed crystal violet was solubilized in 95% ethanol. Absorbance was measured at 570 nm using a microplate spectrophotometer (SpectraMax iD5 Multi-Mode Microplate Reader, Molecular Devices, USA). In parallel, viable bacterial counts were determined by sonicating the skin explants in 0.85% normal saline solution to detach bacteria from the tissue and biofilm, followed by plating on MSA. All experiments were performed in at least quadruplicate across three independent trials.

### 2.13 Statistical analysis

Statistical analyses were conducted using GraphPad Prism version 10. For antibacterial and antibiofilm assays performed *in vitro* and *ex vivo*, bacterial and phage counts were log_10_-transformed prior to analysis. Group comparisons were performed using one-way ANOVA followed by Tukey’s honestly significant difference (HSD) *post hoc* test.

## 3 Results and discussion

### 3.1 Phage morphological and biological characteristics of phage Pelagios

**FIGURE 1:**
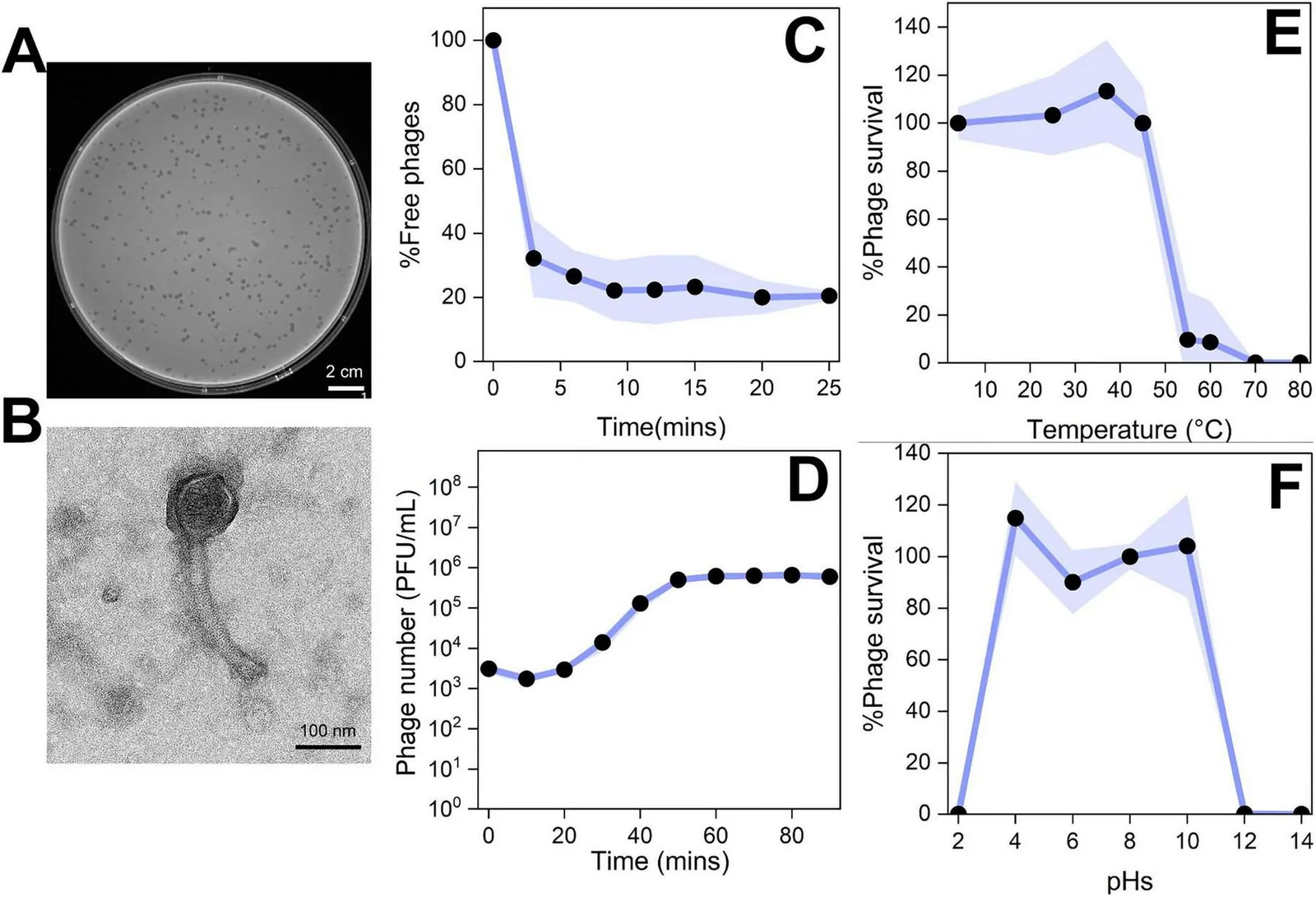
Morphological and biological characteristics of phage Pelagios. **(A)** Small clear plaques of phage Pelagios were formed on the MRSA lawn using 0.35% TSB soft agar and incubated at 37 °C for 16 h. Scale bar equals 2 cm. **(B)** Morphology of phage Pelagios observed by transmission electron microscopy after negative staining with 2% uranyl acetate. Biological characteristics of phage Pelagios. **(C)** Adsorption kinetics of phage Pelagios to the surface of the host strain at a MOI of 0.01. **(D)** One-step growth kinetics in MRSA 40108 at an MOI of 0.01 over 140 min. Phage stability under different pH conditions **(E)** and temperatures **(F)** were assessed after 2 h of incubation. All data **(A–F)** are presented as the mean ± SD of at least four independent experiments.

A methicillin-resistant *Staphylococcus aureus* (MRSA) phage, Pelagios, was successfully isolated from wastewater collected at Songklanarin Hospital, Thailand, using MRSA 40108, isolated from Naradhiwas Rajanagarindra Hospital, Thailand, as the parental host. Wastewater is widely recognized as a rich source of phages due to its high bacterial diversity and ongoing host–phage interactions, which promote the presence of phages capable of infecting clinically relevant pathogenetic bacteria. The Pelagios phage formed small, clear plaques with an average diameter of ∼1.0 ± 0.18 mm (*n* = 50) on the bacterial lawn (Figure 1A), indicating lytic activity. We then examined the morphology of Pelagios using transmission electron microscopy, which revealed a typical head-tail structure comprising an icosahedral head measuring ∼ 82 ± 6 nm in height and 71 ± 4 nm in width (*n* = 12), and a contractile tail measuring ∼213 ± 7 nm in length and 21 ± 2 nm in width (*n* = 12) (Figure 1B). These morphological features indicate that Pelagios belongs to the class Caudoviricetes and is consistent with members of the myovirus morphology group, which is characterized by icosahedral heads and contractile tails (Turner et al., 2023).

To characterize the biological properties of phage Pelagios, the phage adsorption to host cells, replication kinetics, and phage proliferation were investigated using adsorption assay and one-step growth curve analysis. The results demonstrated that the majority of phage Pelagios particles (∼80%) rapidly adsorbed onto the host cells within 15 min post-infection (Figure 1C). Following adsorption, the phage underwent intracellular replication within the host cells for ∼20 min, corresponding to the latent period. At the end of this phase, phage Pelagios lysed the host cells, resulting in the release of ∼102 progeny phages per infected cell (Figure 1D). Phage Pelagios demonstrated stability across a broad range of temperature and pH conditions. Pelagios retained infectivity at temperatures ranging from 4 °C to 40 °C, with optimal activity observed at 37 °C. At 50 °C–60 °C, infectivity was dramatically reduced, whereas the phage was almost completely inactivated at temperatures above 70 °C–80 °C (Figure 1E). Similarly, the phage remained viable at pH 4–10 but completely lost infectivity under highly acidic (pH 2) and strongly alkaline (pH 12–14) conditions (Figure 1F).

To further evaluate the therapeutic potential of phage Pelagios, its host range and efficiency of plating (EOP) were assessed against a panel of clinical *Staphylococcus* spp. strains. The results showed that phage Pelagios exhibited a relatively broad host range, as indicated by its ability to produce clear lysis zones in 7 of the 12 tested clinical *S. aureus* strains, including isolates from urine (MRSA strain 40149), pus (MRSA strain 50017), tissue (MRSA strain 30002), sputum (strain 535896), and corneal ulcer (strains A7-1 and A7-2), as well as the reference strain ATCC 29312 (Table 1). In contrast, it did not infect the other tested *S. epidermidis* and *Staphylococcus* spp. strains. Phage Pelagios displayed high productive infection primarily in selected MRSA clinical isolates, including strains 40149, A7-1, A7-2, and ATCC 29312 (Table 1). These findings highlight the relatively broad host range of phage Pelagios, demonstrating its potent antibacterial activity against a diverse array of clinical MRSA isolates.

### 3.2 Genomic properties and comparative genome analysis of phage Pelagios

**FIGURE 2:**
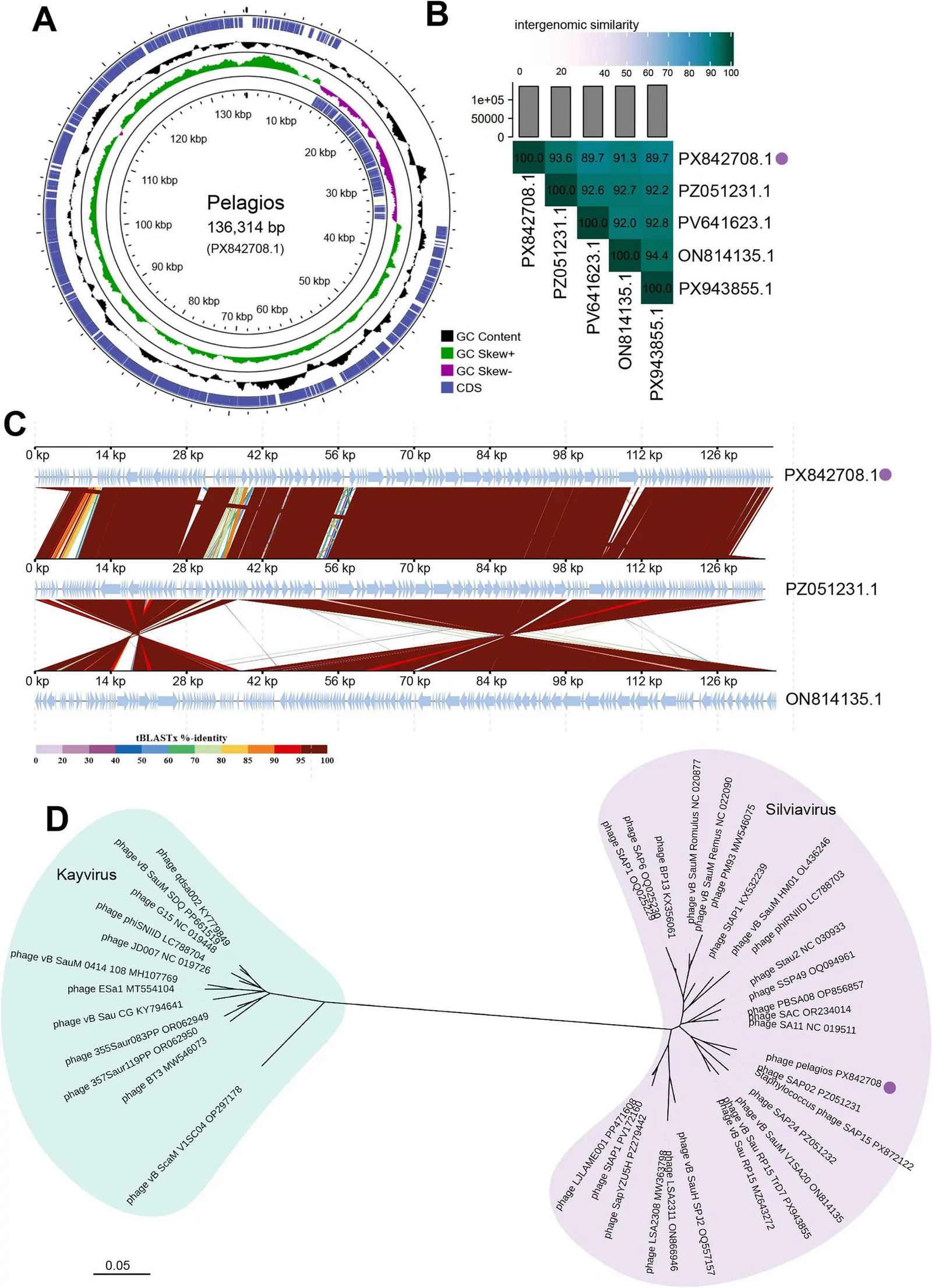
Genome characteristics of phage Pelagios. **(A)** Genome map of Pelagios, which has a 136,314-bp genome comprising 186 ORFs. **(B)** VIRIDIC heatmap showing the intergenomic similarities between Pelagios and related phages, including phage SAP02 (PZ051231.1), phage St1.CM (PV641623.1), phage vB_SauM-V1SA20 (ON814135.1), and phage vB_Sau-RP15-TrD7 (PX943855.1). The numbers indicate pairwise genome similarity values. **(C)** Comparative genome analysis of Pelagios, SAP02, and vB_SauM-V1SA20. Arrows indicate the direction and location of coding sequences, and colored shading lines represent the degree of sequence homology. **(D)** Whole-genome sequence-based phylogenetic tree of Pelagios (purple dot) and selected *Silviavirus* phages. The tree was constructed using the Genome-BLAST Distance Phylogeny (GBDP) method in the VICTOR web service with 100 bootstrap replicates and visualized using iTOL.

Although phage Pelagios demonstrated favorable phenotypic traits, including strong infectivity against several selected clinical *S. aureus* strains, its suitability for therapeutic application also depends on genomic safety. Whole genome sequencing is therefore essential to determine whether the phage encodes undesirable genes associated with antibiotic resistance, bacterial virulence, or lysogeny. To characterize the genomic features of Pelagios, its complete genome was sequenced using the Illumina platform, successfully assembled, and deposited in the NCBI database under accession number PX842708.1. Pelagios harbors a relatively large genome of 136,314 base pairs, encoding a total of 186 ORFs (Figure 2A and Supplementary Table 1). In order to further gain deeper insights into its genetic composition, all predicted ORFs were analyzed using PHASTER and the NCBI database. Of these, 68 ORFs were functionally classified as genes with direct identity to those of Staphylococcus phages (BLASTp, E < 10^–5^), and were associated with DNA replication transcription and translation, DNA metabolism and modification, virion structural and assembly, and bacterial lysis proteins (Supplementary Table 1). The remaining 118 ORFs were annotated as hypothetical proteins with unknown functions. Moreover, Pelagios encodes several genes associated with protection against host restriction systems, including a site-specific DNA methyltransferase (ORF124) and an adenine-specific methyltransferase (ORF127). These enzymes are likely involved in DNA methylation, which protects phage DNA from host restriction enzymes, thereby enhancing phage replication and infection efficiency. Moreover, the presence of mobile genetic element-associated genes, such as a transposase (ORF60) and an HNH endonuclease (ORF135), suggests a potential contribution to genome plasticity. The presence of such elements may reflect the ongoing evolutionary processes and the dynamic relationship between phages and their bacterial hosts.

In addition, despite its myovirus-like morphology, precise taxonomic classification of Pelagios cannot be determined on the basis of morphology alone, as current phage taxonomy relies primarily on genomic criteria. To assess its genetic relationship to other known *Staphylococcus* phages, BLASTn analysis was first performed to identify closely related phages, followed by intergenomic similarity analysis using the Virus Intergenomic Distance Calculator (VIRIDIC). Pelagios showed the highest intergenomic similarity to *Staphylococcus* phage SAP02 (∼94%; PZ051231.1), followed by phages vB_SauM-V1SA20 (Kolenda et al., 2022) (∼91%; ON814135.1), vB_Sau-RP15-TrD7 (∼90%; PX943855.1), and *Staphylococcus* phage St1.CM (∼90%; PV641623.1) (Figure 2B). These values suggest that Pelagios belongs to the genus *Silviavirus* within the family Herelleviridae and may represent a novel species, as its intergenomic similarity remains below the 95% species demarcation threshold. Comparative genomic analysis of Pelagios with the reference phages SAP02 and vB_SauM-V1SA20 revealed a high degree of overall genomic conservation, accompanied by several chromosomal rearrangements and inversions (Figure 2C). Consistent with these observations, phylogenetic tree positioned Pelagios among its closest relatives within the genus *Silviavirus* (Figure 2D).

To assess the safety of phage Pelagios for potential therapeutic applications, its genome was screened for toxin-encoding proteins, virulence factors, and antimicrobial resistance (AMR) genes. Toxin prediction using CSM-toxin, ToxinPred3.0, and DBETH revealed no ORFs consistently classified as toxins (Supplementary Figure 1A); although some ORFs yielded low probability scores, they were ultimately categorized as non-toxins. Furthermore, virulence factor analysis using VFanalyzer (VFDB) and VirulenceFinder 2.0 identified no virulence-associated genes in the Pelagios genome (Supplementary Figure 1B). Similarly, AMR gene screening using ResFinder (v4.7.2) revealed no detectable antibiotic resistance genes (Supplementary Figure 2). The absence of these pathogenic determinants indicates high genomic safety and suggests a low risk of enhancing bacterial pathogenicity. While the presence of mobile genetic element-associated genes, such as a transposase (ORF60) and an HNH endonuclease (ORF135), indicates a potential capacity for genomic plasticity, this does not necessarily increase the risk of mediating harmful horizontal gene transfer (HGT). The safety of a phage in the context of HGT is determined not only by the presence of mobilization machinery but, more critically, by the genetic cargo being transferred. Because our whole-genome sequencing confirmed the absence of known toxins, virulence factors, or antibiotic resistance determinants, the phage lacks the pathogenic ‘payload’ required to pose a risk to the host via horizontal transfer. Thus, the observed genomic plasticity is likely limited to non-pathogenic rearrangements within the phage genome itself, reinforcing its suitability for phage therapy.

### 3.3 Phage Pelagios inhibited MRSA growth both *in vitro* and in an *ex vivo* model and effectively prevented biofilm formation in a dose-independent manner

**FIGURE 3:**
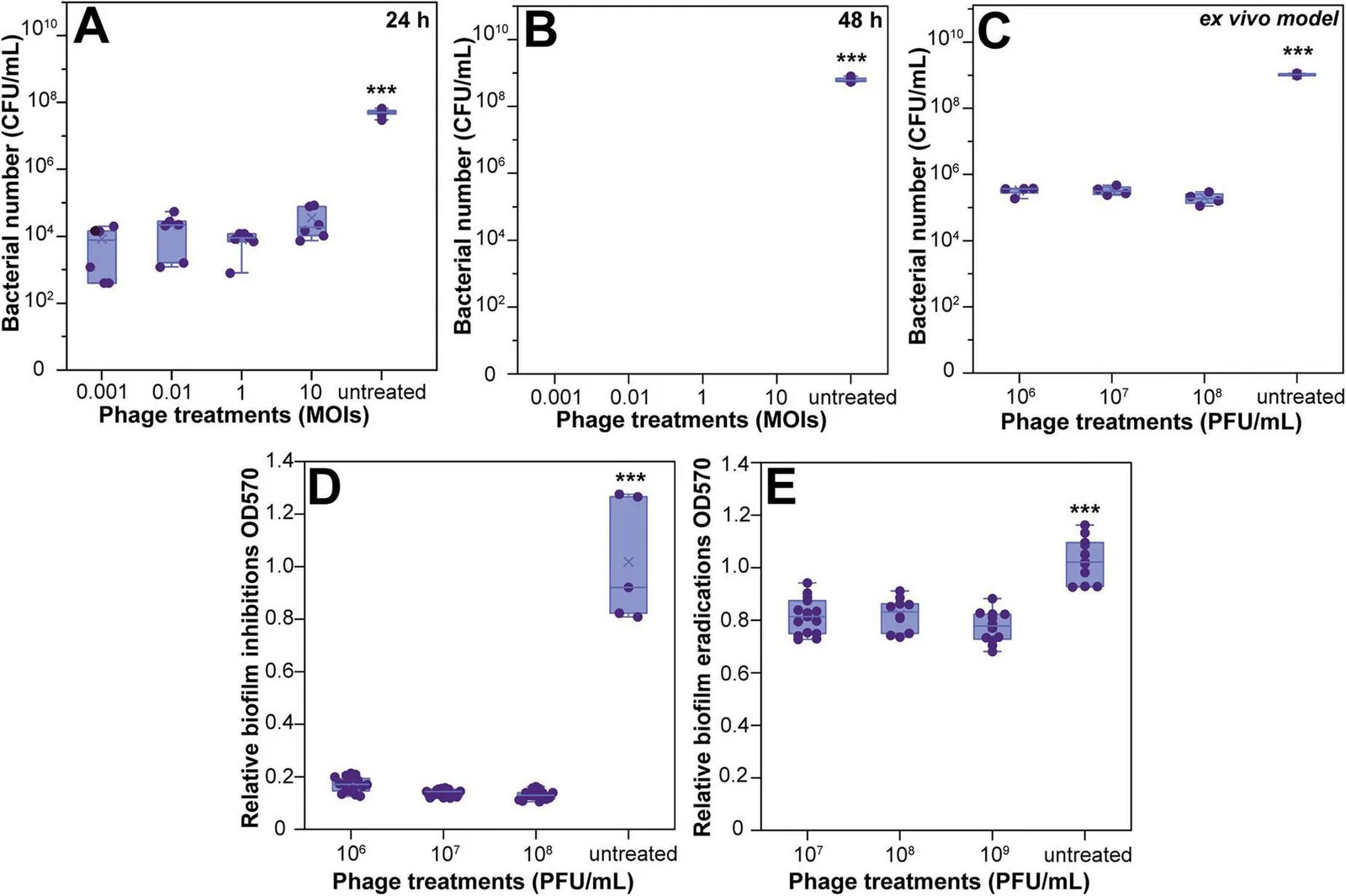
Antibacterial and antibiofilm activities of phage Pelagios *in vitro* and *ex vivo*. Bacterial growth inhibition, measured by viable bacteria, was assessed after treatment with phage Pelagios at different MOIs for 24 h **(A)** and 48 h **(B)**. Antibacterial activity of phage Pelagios in an *ex vivo* wound-infection model was evaluated after 24 h of treatment at different concentrations **(C)**. Biofilm prevention **(D)** and biofilm removal **(E)** activities were evaluated using phage treatments at different concentrations. Biofilm biomass was quantified by crystal violet staining and measured as OD_570_. Each data point represents an independent biological replicate (at least *n* = 5), with averages shown as boxplots. Statistical analysis for panels **A–E** was performed using one-way ANOVA followed by Tukey’s HSD *post hoc* test. Asterisks above groups indicate significant differences (***P ≤ 0.001).

The therapeutic potential of *Silviavirus* phages against *S. aureus* infections has been demonstrated by their broad host range (Kolenda et al., 2022), which enhances pathogen inhibition and confers potent antimicrobial activity both *in vitro* (Lu et al., 2023; André et al., 2025) and *in vivo* against MRSA (Lu et al., 2023; Lerdsittikul et al., 2024). Here, we further evaluated antibacterial efficacy of phage treatment at varying MOIs exposure, together with its capacity for biofilm inhibition and eradication. To assess the bactericidal activity of phage Pelagios, viable MRSA populations were quantified following phage infection at MOIs of 0.001, 0.01, 1, and 10 over 24 and 48 h (Figures 3A, B). At 24 h post-infection, viable MRSA counts in all phage-treated groups were significantly reduced by ∼3–4 log_10_ CFU/mL compared with untreated controls (Figure 3A). Furthermore, at 48 h post-infection, no viable MRSA were detectable in any of the phage-treated groups, irrespective of MOI (Figure 3B), indicating that the bactericidal activity of phage Pelagios was independent of the administered viral dose. The antibacterial effects of phage Pelagios were subsequently evaluated in a wound-infection model using *ex vivo* porcine skin. Viable MRSA counts were significantly reduced by ∼4 log_10_ CFU/mL across all MOIs compared with the untreated control at 24 h post-infection (Figure 3C), indicating a dose-independent antibacterial effect.

Methicillin-resistant *Staphylococcus aureus* frequently colonizes and infects wounds, where biofilm formation facilitates bacterial adherence and tissue invasion, thereby promoting evasion of host immune responses and conferring resistance to antimicrobial therapies (Uberoi et al., 2024). To evaluate the biofilm-inhibitory and biofilm-eradicative potential of phage Pelagios, MRSA biofilms were cultivated on 96-well polystyrene plates for 48 h to permit maturation. The phage suspensions at titers of 10^6^, 10^7^, and 10^8^ PFU/mL were subsequently applied to the biofilms for 24 h, with treatments administered either prior to or following maturation. Crystal violet staining revealed that all phage-treated groups exhibited significantly reduced biofilm biomass compared with the untreated control (Figure 3D), with comparable reductions observed across all phage titers tested. This finding indicates that phage Pelagios effectively inhibited MRSA biofilm formation in a dose-independent manner. However, its biofilm-eradicating efficacy was less pronounced than its preventive effect. Although reductions in biofilm biomass were modest, the treated groups consistently displayed significantly lower biomass relative to the untreated control across all phage titers (Figure 3E). This observation may be attributed to the bactericidal activity of phage Pelagios against MRSA (Figures 3A, B), which consequently reduces biofilm formation, rather than to direct eradication of established biofilms, as comprehensive whole-genome analysis revealed the absence of genes encoding depolymerase activity (Supplementary Table 1), which is typically required for degradation of the biofilm extracellular polymeric matrix (Knecht et al., 2020; Su et al., 2025; Naknaen et al., 2026).

### 3.4 Morphology, skin penetration, and dissolving properties of DDMNs in *ex vivo*

**FIGURE 4:**
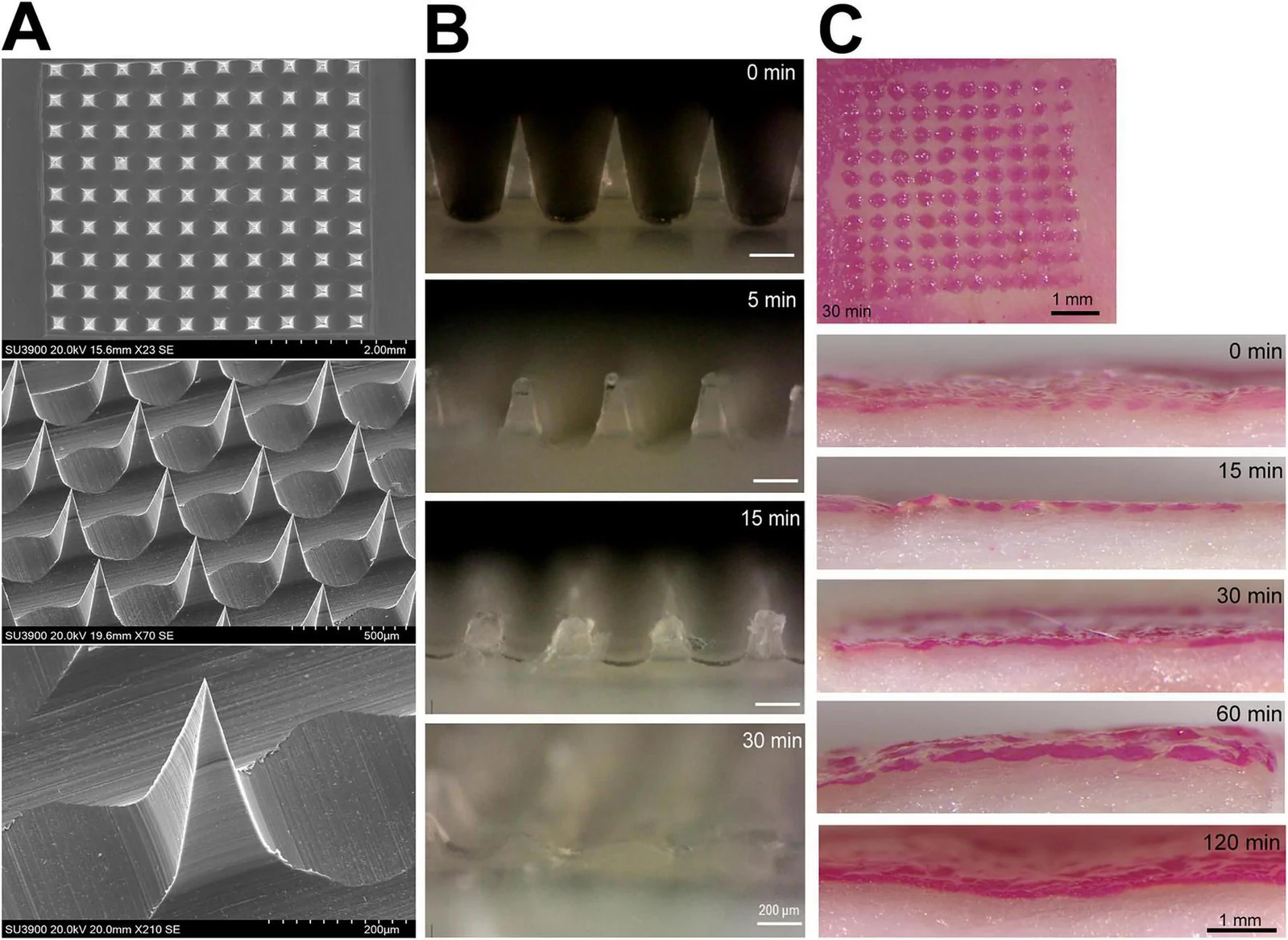
Morphological characterization, skin penetration, and diffusion of DDMNs in porcine ear skin. **(A)** Representative SEM images of DDMNs, showing the top view and magnified fields. **(B)** Dissolving profiles of DDMNs at 5, 15, and 30 min. **(C)** Top-view images of *ex vivo* porcine skin showing the detachment of red dye-spiked DDMNs at 30 min. Cross-sectional and representative stereomicroscopic images of surgically excised porcine ear skin immediately after application of red dye loaded DDMNs at the indicated time points.

Even though phage Pelagios exhibited promising antibacterial activity against planktonic MRSA states, thereby preventing biofilm formation, its efficacy in eradicating established biofilms and in *ex vivo* infection models remained limited. Consequently, advanced phage delivery systems, such as detachable dissolvable microneedles (DDMNs) technology (Sawutdeechaikul et al., 2021; Chen et al., 2025), have been proposed to enhance therapeutic efficacy and stability in wound infections. This delivery platform could improve the efficiency of transdermal phage administration while preserving phage viability, thereby enabling targeted delivery to the pathogen within the biofilm matrix and increasing overall therapeutic efficacy (Efendi et al., 2025; Javed et al., 2026). We then modified the detachable dissolvable microneedles developed in our previous study (Efendi et al., 2025) to deliver phage Pelagios and thereby may overcome these limitations. DDMN patches, each consisting of 100 tetragonal pyramidal needles (300 × 300 μm base and 650 μm needle height), were initially prepared from 17% hyaluronic acid (HA) and 3% gelatin. The HA solution was sterilized by autoclaving prior to fabrication following our previous protocol (Efendi et al., 2025). Although autoclaving may induce partial degradation of HA, previous studies have demonstrated that this fabrication process produces DDMNs with satisfactory mechanical properties, skin penetration capability, dissolution behavior, and phage stability (Efendi et al., 2025). Consistent with these findings, the DDMNs fabricated in the present study exhibited uniform morphology with sharp, well-defined needles. The morphology was further examined using SEM, which showed that the front and side views of the DDMNs appeared solid and sharp (Figure 4A), with a needle height of ∼ 450 ± 22 μm (*n* = 35), indicating successful and complete casting. Nevertheless, we acknowledge that we did not directly evaluate the effects of autoclaving on the molecular weight or rheological properties of HA. Future studies incorporating gel permeation chromatography and rheological analyses would provide further insight into the influence of thermal sterilization on HA and its potential impact on microneedle performance.

As the incomplete transfer of needles from DDMNs into the skin has long been recognized as a limitation, conventional DDMN patches must typically remain on the skin for a sufficient period, usually at least 2 h, to allow the needle portion to dissolve within the skin tissue before the baseplate is removed. Therefore, we focused on developing detachable DDMN patches capable of achieving complete needle deposition into the skin, and their dissolution performance was subsequently evaluated *ex vivo*. The dissolution test confirmed complete dissolution of all DDMNs within 30 min in porcine ear skin (Figure 4B). To enable visualization of the dissolving microneedle state and drug release behavior, red dye (Rhodamine B) was incorporated into the polymer solution during DDMN fabrication. Successful detachment was achieved in all DDMNs, with no residual needles remaining on the backing sheet after removal. Clear red spots were observed in the top-view images of the skin, corresponding precisely to the needle pattern on the patch (Figure 4B). Furthermore, DDMNs were applied to porcine ear skin, and cross-sectional analysis was performed on the skin tissues at various time points post-application. As shown in Figure 4C, at 0 min (immediately after DDMN application), an array of red spots was visible, with the spacing between spots matching the gaps between needles on the DDMNs. Each red spot extended from the stratum corneum to a depth of ∼0.02–0.05 mm (epidermis and upper dermis). At 15 min, the red dye began to diffuse, nearly closing the gaps observed at 0 min and then after 30 min, the dye continued to spread horizontally. By 60 and 120 min, the red dye covered a depth of ∼0.2–0.6 mm, indicating successful dissolution and drug release from the DDMNs. However, it should be noted that the diffusion rate of DDMNs may differ among skin types, as DDMNs penetrated the epidermis and upper dermis of porcine ear skin faster than rat skin, likely reflecting faster diffusion due to lower dermal lipid content (Sawutdeechaikul et al., 2021). *Ex vivo* porcine skin is widely accepted as a surrogate for human skin because it exhibits comparable drug permeation and skin retention profiles, making it a suitable model for evaluating microneedle-mediated delivery (Herkenne et al., 2006; Brighenti et al., 2025). Nevertheless, the absence of blood circulation, immune responses, and wound-healing processes in *ex vivo* tissue remains a limitation, and future *in vivo* studies are needed to validate the pharmacokinetics and therapeutic efficacy of phage-loaded DDMNs before clinical translation.

### 3.5 Improved antibacterial and biofilm eradication by PDDMNs in *ex vivo* skin tissue

**FIGURE 5:**
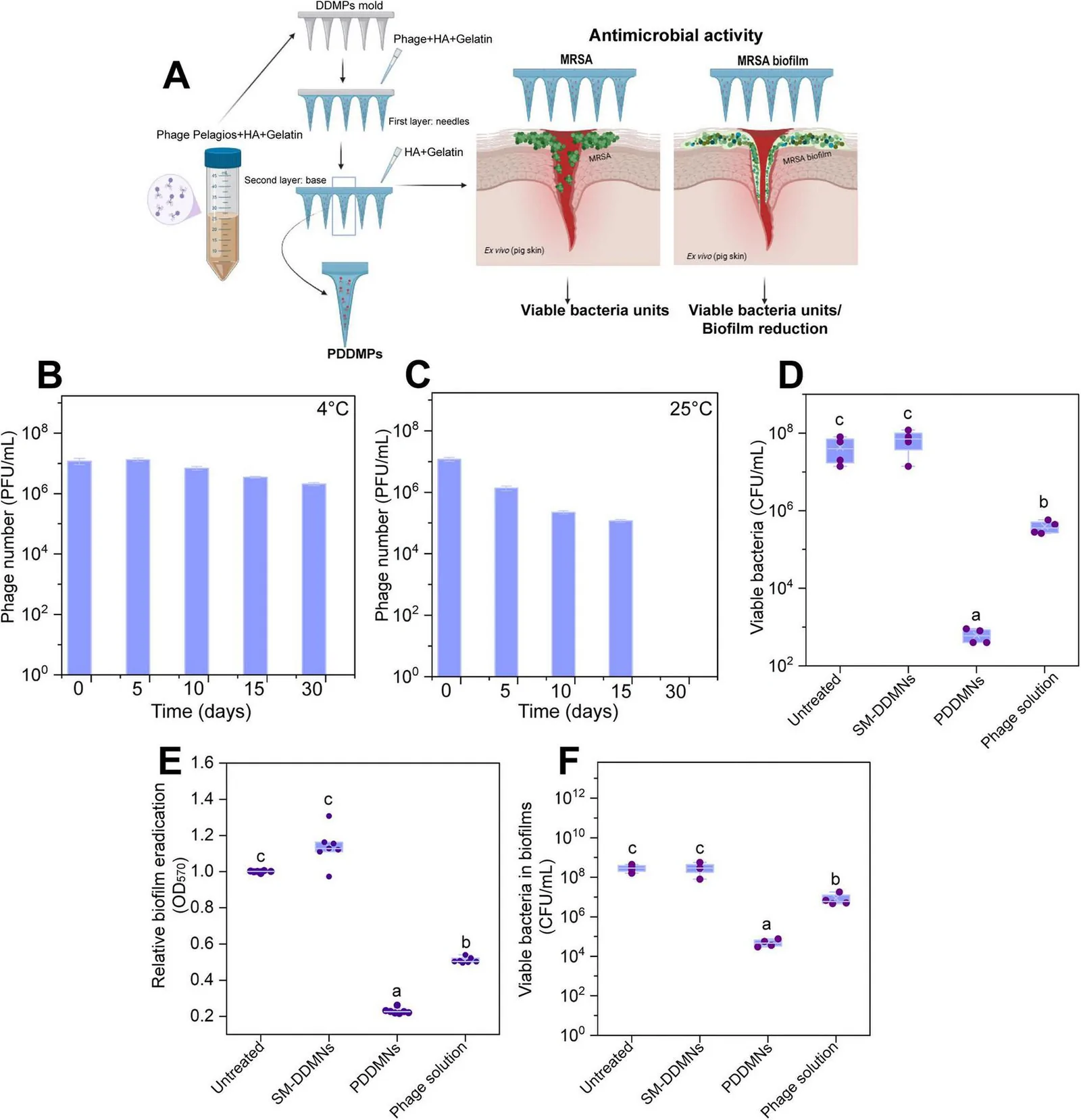
Bactericidal activity and biofilm eradication of PDDMNs against MRSA on porcine ear skin. **(A)** Schematic illustration of the preparation of phage-loaded DDMNs generated using BioRender. **(B,C)** Stability of phage Pelagios when embedded in DDMNs and stored at 4 °C **(B)** and 25 °C **(C)**. **(D–F)** Ability of phage-loaded DDMNs and phage suspensions to eliminate MRSA **(D)** and eradicate its biofilm **(E)**, together with viable bacterial counts **(F)**. All data **(B–F)** are presented as the mean ± SD of at least four independent experiments. One-way ANOVA with Tukey’s *post-hoc* test was used to determine significant differences; letters indicate significant differences at *p* < 0.05.

With our goal to use DDMNs to deliver phage Pelagios in *ex vivo* porcine ear skin infection models, phage-loaded DDMNs (PDDMNs) were fabricated (Figure 5A). The stability of viable phages incorporated into DDMNs (initial titer ∼ 1 × 10^7^ PFU/mL) was evaluated during storage at 4 °C and 25 °C. Phage Pelagios exhibited markedly greater stability at 4 °C, retaining a titer of ∼ 1 × 10^6^ PFU/mL after 30 days (Figure 5B). In contrast, the titer at 25 °C declined steadily and fell below the limit of detection by day 30 (Figure 5C), indicating that storage at 4 °C effectively preserves phage viability within the dissolving HA-gelatin microneedles. Comparable studies have reported enhanced phage stability in microneedle or hydrogel formulations relative to free suspensions; for example, *Pseudomonas* phage-loaded microneedles remained stable for 28 days at 4 °C (Efendi et al., 2025), while polyvinyl alcohol microneedles exhibited ∼1 log_10_ and ∼2 log_10_ reductions after 30 days at 4 °C and 25 °C, respectively (Sillankorva et al., 2022), and HA–alginate–chitosan hydrogels have been shown to maintain phage infectivity at 4 °C (Rotman et al., 2023). Previous studies have demonstrated that incorporation of phages into the HA–gelatin matrix induces a shift in the endothermic peak, as measured by differential scanning calorimetry (Moghtader et al., 2024; Efendi et al., 2025). This shift is consistent with the formation of new intermolecular interactions between phage proteins and the surrounding polymer network. These interactions are proposed to restrict protein conformational changes and thereby enhance phage thermal stability, consistent with prior observations that electrostatic interactions between gelatin and partially unfolded proteins can prevent aggregation (Thyagarajapuram et al., 2007). Nevertheless, several limitations should be acknowledged. Although the HA-gelatin DDMNs effectively preserved phage viability for up to 30 days at 4 °C, longer-term stability under different storage conditions remains to be investigated to establish the shelf life of the formulation. Furthermore, the biocompatibility of the HA-gelatin matrix toward skin cells, the potential immunogenicity of phage Pelagios following microneedle-mediated delivery, and the scalability and sterility validation of the manufacturing process were not evaluated in the present study. Addressing these aspects, together with *in vivo* efficacy and safety studies, will be essential for the further development and clinical translation of this phage-loaded DDMN platform.

To evaluate the antimicrobial efficacy of phage Pelagios-loaded DDMNs against both planktonic and biofilm-associated MRSA, viable bacterial counts in infected porcine ear skin were quantified following topical application of PDDMNs and compared with those obtained after treatment with a free phage suspension. We hypothesized that DDMNs would improve local phage delivery by successfully penetrating the porcine skin and depositing the phages directly into the infected tissue (Figure 4C). Treatment with phage Pelagios-loaded DDMNs significantly reduced viable planktonic MRSA by approximately 3 log_10_ CFU/mL compared with the free phage suspension after 24 h (*p* < 0.05; Figure 5D), whereas the control and blank DDMNs showed no significant antibacterial activity (*p* > 0.05). Similarly, PDDMNs significantly enhanced biofilm eradication (*p* < 0.05; Figure 5E), reducing viable biofilm-associated MRSA by approximately 2 log_10_ CFU/mL compared with the free phage suspension (*p* < 0.05; Figure 5F). These findings suggest that direct deposition of phages into the skin by DDMNs may improve phage access to bacteria within infected tissue and biofilm structures, resulting in greater antibacterial efficacy than topical phage suspension. However, quantitative phage release kinetics and diffusion within skin were not evaluated in the present study. Therefore, the proposed mechanism of enhanced local phage delivery remains inferential and should be confirmed by future studies investigating phage release, tissue distribution, and biodistribution following DDMN application.

## 4 Conclusion

Our study demonstrates that phage Pelagios is a promising lytic phage with therapeutic potential against multiple clinical MRSA isolates. Pelagios exhibited myovirus-like morphology, rapid adsorption, a short latent period, a high burst size, and broad stability across physiologically relevant temperature and pH ranges. Whole-genome sequencing confirmed its genomic safety, with no detectable toxin genes, virulence factors, antimicrobial resistance determinants, or lysogeny-associated genes. Comparative genomic and phylogenetic analyses further classified Pelagios within the genus *Silviavirus* (family Herelleviridae), suggesting that it represents a novel species. Pelagios effectively inhibited planktonic MRSA growth both *in vitro* and in an *ex vivo* porcine skin infection model and prevented biofilm formation, although its activity against established biofilms was limited, likely due to the absence of depolymerase-associated genes. To improve local phage administration, DDMNs were developed as a transdermal delivery platform. The DDMNs exhibited efficient skin penetration, rapid dissolution, complete needle detachment, and preserved phage viability during storage at 4 °C for up to 30 days. Moreover, phage-loaded DDMNs demonstrated significantly greater antibacterial and antibiofilm activity than free phage suspension in the *ex vivo* porcine skin model, suggesting improved local phage delivery. Although these findings support the potential of phage-loaded DDMNs as a promising strategy for the treatment of MRSA-associated wound and biofilm-related infections, further studies are required to evaluate long-term formulation stability, phage release kinetics, pharmacokinetics, biocompatibility, immunogenicity, scalable sterile manufacturing, and *in vivo* therapeutic efficacy before clinical translation.

## Data Availability

The datasets presented in this study can be found in online repositories. The names of the repository/repositories and accession number(s) can be found in the article/Supplementary material.

## Footnotes

1
https://usegalaxy.eu/

2
https://phaster.ca/

3
https://rhea.icbm.uni-oldenburg.de/VIRIDIC/

4
https://www.genome.jp/digalign/

5
https://ggdc.dsmz.de/victor.php

6
https://itol.embl.de/login.cgi

7
https://genepi.food.dtu.dk/resfinder

8
https://biosig.lab.uq.edu.au/csm_toxin/

9
https://webs.iiitd.edu.in/raghava/toxinpred3/

10
https://www.mgc.ac.cn/VFs/main.htm

11
https://cge.food.dtu.dk/services/VirulenceFinder/

## References

[B1] Alves D. R. Gaudion A. Bean J. E. Perez Esteban P. Arnot T. C. Harper D. R. et al. (2014). Combined use of bacteriophage k and a novel bacteriophage to reduce *Staphylococcus aureus* biofilm formation. *Appl. Environ. Microbiol.* 80 6694–6703. 10.1128/AEM.01789-14 25149517 PMC4249044

[B2] André C. Medina M. Kolenda C. Blazière L. Helluin E. Resch G. et al. (2025). In Vitro activity of bacteriophages against ocular methicillin-resistant *S. aureus* isolates collected in the US. *Ophthalmol. Ther.* 14 897–909. 10.1007/s40123-025-01113-2 40072828 PMC12006626

[B3] Azevedo M. M. Pina-Vaz C. Rodrigues A. G. (2022). The role of phage therapy in burn wound infections management: Advantages and pitfalls. *J. Burn Care Res.* 43 336–342. 10.1093/jbcr/irab175 34523679

[B4] Bessa L. J. Fazii P. Di Giulio M. Cellini L. (2015). Bacterial isolates from infected wounds and their antibiotic susceptibility pattern: Some remarks about wound infection. *Intern. Wound J.* 12 47–52. 10.1111/iwj.12049 23433007 PMC7950398

[B5] Brighenti M. D. S. Montanheri L. R. D. S. Duque M. D. Andreo-Filho N. Lopes P. S. Garcia M. T. J. et al. (2025). *In Vitro* drug release and *Ex Vivo* dermal drug permeation studies of selected commercial benzoyl peroxide topical formulations: Correlation between human and porcine skin models. *Mol. Pharmaceut.* 22 1365–1372. 10.1021/acs.molpharmaceut.4c01058 39899465 PMC11881039

[B6] Chen K. Sun X. Liu Y. Li S. Meng D. (2025). Advances in clinical applications of microneedle. *Front. Pharmacol.* 16:1607210. 10.3389/fphar.2025.1607210 40641999 PMC12242436

[B7] Efendi A. Naknaen A. Ampawa S. Miengmern N. Chaikeeratisak V. Wanichwecharungruang S. (2025). Detachable dissolvable microneedles maintain the viability of phage cocktail and effectively disrupt *Pseudomonas aeruginosa* biofilm. *J. Drug Deliv. Sci. Technol.* 104:106527. 10.1016/j.jddst.2024.106527

[B8] Herkenne C. Naik A. Kalia Y. N. Hadgraft J. Guy R. H. (2006). Pig ear skin ex Vivo as a model for in Vivo Dermatopharmacokinetic studies in man. *Pharm. Res.* 23 1850–1856. 10.1007/s11095-006-9011-8 16841197

[B9] Jamaledin R. Di Natale C. Onesto V. Taraghdari Z. Zare E. Makvandi P. et al. (2020a). Progress in microneedle-mediated protein delivery. *JCM* 9:542. 10.3390/jcm9020542 32079212 PMC7073601

[B10] Jamaledin R. Yiu C. K. Y. Zare E. N. Niu L. Vecchione R. Chen G. et al. (2020b). Advances in antimicrobial microneedle patches for combating infections. *Adv. Mater.* 32:2002129. 10.1002/adma.202002129 32602146

[B11] James G. A. Swogger E. Wolcott R. Pulcini E. deLancey Secor P. et al. (2008). Biofilms in chronic wounds. *Wound Repair Regenerat.* 16 37–44. 10.1111/j.1524-475X.2007.00321.x 18086294

[B12] Javed S. Ruggiero V. Falcone G. Del Gaudio P. Aquino R. P. Auriemma G. et al. (2026). Breaking multidrug resistance: Rediscovering phage therapy and delivery strategies. *J. Drug Deliv. Sci. Technol.* 120:108252. 10.1016/j.jddst.2026.108252

[B13] Knecht L. E. Veljkovic M. Fieseler L. (2020). Diversity and function of phage encoded depolymerases. *Front. Microbiol.* 10:2949. 10.3389/fmicb.2019.02949 31998258 PMC6966330

[B14] Kolenda C. Medina M. Bonhomme M. Laumay F. Roussel-Gaillard T. Martins-Simoes P. et al. (2022). Phage therapy against *Staphylococcus aureus*: Selection and optimization of production protocols of novel broad-spectrum silviavirus phages. *Pharmaceutics* 14:1885. 10.3390/pharmaceutics14091885 36145633 PMC9503876

[B15] Kortright K. E. Chan B. K. Koff J. L. Turner P. E. (2019). Phage therapy: A renewed approach to combat antibiotic-resistant bacteria. *Cell Host Microbe* 25 219–232. 10.1016/j.chom.2019.01.014 30763536

[B16] Kropinski A. M. (2018). “Practical Advice on the One-Step Growth Curve,” in *Bacteriophages*, eds Clokie M. R. J. Kropinski A. M. Lavigne R. (New York, NY: Springer New York), 41–47. 10.1007/978-1-4939-7343-9_3 29134585

[B17] Lerdsittikul V. Apiratwarrasakul S. Atithep T. Withatanung P. Indrawattana N. Pumirat P. et al. (2024). Isolation and characterisation of a novel *Silviavirus bacteriophage* promising antimicrobial agent against methicillin-resistant *Staphylococcus aureus* infections. *Sci. Rep.* 14:9251. 10.1038/s41598-024-59903-w 38649443 PMC11035597

[B18] Li W. Terry R. N. Tang J. Feng M. R. Schwendeman S. P. Prausnitz M. R. (2019). Rapidly separable microneedle patch for the sustained release of a contraceptive. *Nat. Biomed. Eng.* 3 220–229. 10.1038/s41551-018-0337-4 30948808

[B19] Lu Y. Lu Y. Li B. Liu J. Wang L. Zhang L. et al. (2023). StAP1 phage: An effective tool for treating methicillin-resistant Staphylococcus aureus infections. *Front. Microbiol.* 14:1267786. 10.3389/fmicb.2023.1267786 37840707 PMC10570516

[B20] Luo J. Liu M. Ai W. Zheng X. Liu S. Huang K. et al. (2024). Synergy of lytic phage pB23 and meropenem combination against carbapenem-resistant *Acinetobacter baumannii*. *Antimicrob. Agents Chemother.* 68:e0044824. 10.1128/aac.00448-24 38742904 PMC11620502

[B21] Moghtader F. Solakoglu S. Piskin E. (2024). Alginate- and chitosan-modified gelatin hydrogel microbeads for delivery of *E. coli* phages. *Gels* 10:244. 10.3390/gels10040244 38667663 PMC11049077

[B22] Naknaen A. Rueangrit A. Sudhikaran W. Chukamnerd A. Chumtong S. Chusri S. et al. (2026). Synergistic effects of halo-plaque forming phage cocktails against polymicrobial *Acinetobacter baumannii* and *Staphylococcus aureus*. *Microbiol. Spectr.* 14:e0400925. 10.1128/spectrum.04009-25 42059665 PMC13227992

[B23] Naknaen A. Samernate T. Saeju P. Nonejuie P. Chaikeeratisak V. (2024). Nucleus-forming jumbophage PhiKZ therapeutically outcompetes non-nucleus-forming jumbophage Callisto. *iScience* 27:109790. 10.1016/j.isci.2024.109790 38726363 PMC11079468

[B24] Negut I. Grumezescu V. Grumezescu A. M. (2018). Treatment strategies for infected wounds. *Molecules* 23:2392. 10.3390/molecules23092392 30231567 PMC6225154

[B25] O’Flaherty S. Ross R. P. Meaney W. Fitzgerald G. F. Elbreki M. F. Coffey A. (2005). Potential of the polyvalent Anti- *Staphylococcus* bacteriophage K for control of antibiotic-resistant *Staphylococci* from hospitals. *Appl. Environ. Microbiol.* 71 1836–1842. 10.1128/AEM.71.4.1836-1842.2005 15812009 PMC1082512

[B26] Phoka T. Thanuthanakhun N. Visitchanakun P. Dueanphen N. Wanichwecharungruang N. Leelahavanichkul A. et al. (2023). Detachable-dissolvable-microneedle as a potent subunit vaccine delivery device that requires no cold-chain. *Vaccine: X* 15:100398. 10.1016/j.jvacx.2023.100398 37920235 PMC10618702

[B27] Plumet L. Morsli M. Ahmad-Mansour N. Clavijo-Coppens F. Berry L. Sotto A. et al. (2023). Isolation and characterization of new bacteriophages against *Staphylococcal* clinical isolates from diabetic foot ulcers. *Viruses* 15:2287. 10.3390/v15122287 38140529 PMC10747802

[B28] Prausnitz M. R. Gomaa Y. Li W. (2019). Microneedle patch drug delivery in the gut. *Nat. Med.* 25 1471–1472. 10.1038/s41591-019-0606-0 31591600

[B29] Rotman S. G. Post V. Foster A. L. Lavigne R. Wagemans J. Trampuz A. et al. (2023). Alginate chitosan microbeads and thermos-responsive hyaluronic acid hydrogel for phage delivery. *J. Drug Deliv. Sci. Technol.* 79:103991. 10.1016/j.jddst.2022.103991

[B30] Sawutdeechaikul P. Kanokrungsee S. Sahaspot T. Thadvibun K. Banlunara W. Limcharoen B. et al. (2021). Detachable dissolvable microneedles: Intra-epidermal and intradermal diffusion, effect on skin surface, and application in hyperpigmentation treatment. *Sci. Rep.* 11:24114. 10.1038/s41598-021-03503-5 34916571 PMC8677736

[B31] Sen C. K. Gordillo G. M. Roy S. Kirsner R. Lambert L. Hunt T. K. et al. (2009). Human skin wounds: A major and snowballing threat to public health and the economy. *Wound Repair Regenerat.* 17 763–771. 10.1111/j.1524-475X.2009.00543.x 19903300 PMC2810192

[B32] Serra R. Grande R. Butrico L. Rossi A. Settimio U. F. Caroleo B. et al. (2015). Chronic wound infections: The role of *Pseudomonas aeruginosa* and *Staphylococcus aureus*. *Exp. Rev. Anti-infect. Therapy* 13 605–613. 10.1586/14787210.2015.1023291 25746414

[B33] Shan J. Wu X. Che J. Gan J. Zhao Y. (2024). Reactive microneedle patches with antibacterial and dead bacteria-trapping abilities for skin infection treatment. *Adv. Sci.* 11:2309622. 10.1002/advs.202309622 38582511 PMC11186059

[B34] Sillankorva S. Pires L. Pastrana L. M. Bañobre-López M. (2022). Antibiofilm efficacy of the *Pseudomonas aeruginosa* Pbunavirus vB_PaeM-SMS29 loaded onto dissolving polyvinyl alcohol microneedles. *Viruses* 14:964. 10.3390/v14050964 35632706 PMC9143888

[B35] Su J. Tan Y. Liu S. Zou H. Huang X. Chen S. et al. (2025). Characterization of a novel lytic phage vB_AbaM_AB4P2 encoding depolymerase and its application in eliminating biofilms formed by *Acinetobacter baumannii*. *BMC Microbiol.* 25:123. 10.1186/s12866-025-03854-3 40057696 PMC11889872

[B36] Suludere M. A. Öz O. K. Rogers L. C. Wukich D. K. Malone M. Lavery L. A. (2024). MRSA infection, re-infection and clinical outcomes in diabetic foot infections. *Wound Repair Regenerat.* 32 377–383. 10.1111/wrr.13164 38419162

[B37] Thyagarajapuram N. Olsen D. Middaugh C. R. (2007). Stabilization of proteins by recombinant human gelatins. *J. Pharmaceut. Sci.* 96 3304–3315. 10.1002/jps.20980 17542020

[B38] Turner D. Shkoporov A. N. Lood C. Millard A. D. Dutilh B. E. Alfenas-Zerbini P. et al. (2023). Abolishment of morphology-based taxa and change to binomial species names: 2022 taxonomy update of the ICTV bacterial viruses subcommittee. *Arch. Virol.* 168:74. 10.1007/s00705-022-05694-2 36683075 PMC9868039

[B39] Uberoi A. McCready-Vangi A. Grice E. A. (2024). The wound microbiota: Microbial mechanisms of impaired wound healing and infection. *Nat. Rev. Microbiol.* 22 507–521. 10.1038/s41579-024-01035-z 38575708

[B40] Zhao G. Usui M. L. Lippman S. I. James G. A. Stewart P. S. Fleckman P. et al. (2013). Biofilms and inflammation in chronic wounds. *Adv. Wound Care* 2 389–399. 10.1089/wound.2012.0381 24527355 PMC3763221

