# Supplementary material for: Detachable dissolving microneedles loaded with Silviavirus phage Pelagios enhance antibacterial efficacy against methicillin resistant Staphylococcus aureus in ex vivo porcine skin
Source: Front Microbiol. 2026 Aug 21;17:1928352. doi: 10.3389/fmicb.2026.1928352 (PMC13541741; doi:10.3389/fmicb.2026.1928352)
Supplement: Supplementary file 1 [file Data_Sheet_1.pdf]

Supplementary Material

Table S1 List of annotated proteins from ORFs in Pelagios genome

ORF	Predicted function	Direction	Start	Stop	Size (nt)	Size (aa)	Coverage	similarity	NCBI Sequence Hits	E-value
1	nuclease	+	665	1066	399	133	100%	77.44%	[Staphylococcus phage SAPYZU_15]	4.00E-62
2	hypothetical protein	+	1192	1401	207	69	100%	94.20%	[Staphylococcus phage Koomba-kaat_1]	2.00E-39
3	putative nuclease	+	1449	1739	288	96	56%	98.15%	[Staphylococcus phage KSAP7]	1.00E-28
4	hypothetical protein	+	1899	2138	237	79	100%	93.67%	[Staphylococcus phage CIFT_MFB_MRSA12]	4.00E-42
5	hypothetical protein	+	2139	2339	198	66	100%	96.97%	[Staphylococcus phage SAC]	2.00E-32
6	hypothetical protein	+	2545	2883	336	112	100%	94.64%	[Staphylococcus phage qdsa001]	5.00E-68
7	hypothetical protein	+	3018	3356	336	112	100%	95.54%	[Staphylococcus phage SAC]	7.00E-70
8	hypothetical protein	+	3356	3664	306	102	100%	97.06%	[Staphylococcus phage vB_SauM-HM01]	1.00E-65
9	membrane protein	+	3815	4216	399	133	100%	96.24%	[Staphylococcus phage SA11]	1.00E-85
10	hypothetical protein	+	4242	4601	357	119	100%	97.48%	[Staphylococcus phage vB_SauM-V1SA20]	9.00E-79
11	hypothetical protein	+	4621	5358	735	245	100%	96.33%	[Staphylococcus phage vB_SauM-V1SA22]	2.00E-166
12	hypothetical protein	+	5404	5613	207	69	100%	98.55%	[Staphylococcus phage vB_SauM_Remus]	4.00E-40
13	hypothetical protein	+	5616	5924	306	102	100%	99.02%	[Staphylococcus phage Koomba-kaat_1]	4.00E-65
14	hypothetical protein	+	6051	6332	279	93	100%	93.55%	[Staphylococcus phage vB_SauM-V1SA22]	5.00E-57
15	hypothetical protein	+	7079	7486	405	135	100%	97.04%	[Staphylococcus phage St1.CM]	2.00E-88
16	hypothetical protein	+	8097	8411	312	104	100%	98.06%	[Silviavirus remus]	8.00E-65
17	hypothetical protein	+	8484	8639	153	51	100%	92.16%	[Staphylococcus phage vB_SauM_VL10]	5.00E-27
18	hypothetical protein	+	8641	8874	231	77	100%	100.00%	[Staphylococcus phage vB_SauM-V1SA20]	2.00E-49
19	hypothetical protein	+	8956	9102	144	48	100%	100.00%	[Staphylococcus phage vB_SauM-V1SA20]	9.00E-25
20	TreN-like membrane protein	+	9157	9303	144	48	100%	93.75%	[Staphylococcus phage PG-2021_5]	4.00E-16
21	terminal repeat-encoded protein	+	9504	9845	339	113	76%	60.92%	[Staphylococcus phage UHP46]	2.00E-29
22	hypothetical protein	+	10000	10689	687	229	100%	99.56%	[Staphylococcus phage phiRNIID]	3.00E-156
23	hypothetical protein	-	11456	11851	393	131	100%	94.66%	[Staphylococcus phage vB_SauM_Romulus]	1.00E-83
24	hypothetical protein	-	11869	12108	237	79	100%	87.34%	[Staphylococcus phage phiRNIID]	2.00E-43
25	YpoX protein I	-	12111	12497	384	128	97%	91.13%	[Staphylococcus phage vB_SauM_Romulus]	3.00E-77
26	hypothetical protein	-	12497	12691	192	64	100%	85.94%	[Staphylococcus aureus]	6.00E-30
27	hypothetical protein	-	12688	13092	402	134	9%	97.74%	[Staphylococcus phage vB_Sau-RP15]	2.00E-91
28	hypothetical protein	-	13104	13634	528	176	100%	97.73%	[Staphylococcus phage vB_SauM-V1SA20]	6.00E-120
29	hypothetical protein	-	13760	14320	558	186	100%	99.46%	[Staphylococcus phage StAP1]	1.00E-131
30	major tail protein	-	14337	14660	321	107	100%	96.26%	[Staphylococcus phage SA11]	3.00E-67
31	hypothetical protein	-	14750	14974	222	74	100%	97.30%	[Staphylococcus phage vB_SauM_Romulus]	2.00E-42
32	hypothetical protein	-	14967	15752	783	261	100%	99.23%	[Staphylococcus phage vB_SauM-V1SA19]	0

Table S1 List of annotated proteins from ORFs in Pelagios genome (cont.)

ORF	Predicted function	Direction	Start	Stop	Size (nt)	Size (aa)	Coverage	similarity	NCBI Sequence Hits	E-value
33	endonuclease	-	16136	16498	360	120	100%	99.17%	[Staphylococcus phage SAC]	2.00E-80
35	autotransporter adhesin	-	18995	19735	738	246	100%	91.87%	[Staphylococcus phage phiIPLA-LAVI]	6.00E-153
36	tail fiber protein	-	19951	20322	369	123	98%	74.17%	[Staphylococcus phage J1P1]	2.00E-56
37	RinB-like transcriptional activator	-	20390	20530	138	46	72%	90.91%	[Staphylococcus phage SAP_01]	1.00E-05
38	hypothetical protein	-	20527	20850	321	107	100%	97.20%	[Staphylococcus phage Stau2]	3.00E-69
39	hypothetical protein	-	20837	21253	414	138	100%	97.10%	[Staphylococcus phage vB_Sau-RP15]	4.00E-92
40	hypothetical protein	-	21372	21557	183	61	100%	96.72%	[Staphylococcus phage SA11]	2.00E-32
41	hypothetical protein	-	21557	21718	159	53	96%	88.24%	[Staphylococcus aureus]	1.00E-22
42	hypothetical protein	-	21718	23232	1512	504	100%	94.84%	[Staphylococcus phage vB_Sau-RP15]	0
43	hypothetical protein	-	23255	23758	501	167	96%	95.03%	[Staphylococcus phage StAP1]	8.00E-100
44	hypothetical protein	-	23837	24100	261	87	100%	97.70%	[Staphylococcus phage KSAP7]	2.00E-51
45	LysM motif protein	-	24114	24287	171	57	100%	98.25%	[Staphylococcus phage Koomba-kaat_1]	1.00E-31
46	hypothetical protein	-	24262	24885	621	207	100%	97.10%	[Staphylococcus phage vB_SauM_Romulus]	1.00E-138
47	nucleosidedeoxy ribosyltransferase	-	24878	25471	591	197	100%	87.82%	[Staphylococcus phage LJLAME001]	5.00E-128
48	hypothetical protein	-	25546	25962	414	138	99%	92.70%	[Staphylococcus phage vB_SauM-V1SA22]	2.00E-87
49	hypothetical protein	-	25904	26158	252	84	100%	98.81%	[Staphylococcus phage CIFT_MFB_MRSA12]	1.00E-52
50	membrane protein	-	26124	26387	261	87	99%	68.60%	[Staphylococcus phage CF5]	4.00E-31
51	ribonuclease H	-	26732	26824	90	30	100%	100.00%	[Staphylococcus phage vB_SauM-V1SA20]	7.00E-12
52	hypothetical protein	-	26817	27005	186	62	100%	98.39%	[Staphylococcus phage vB_SauH_SPJ2]	5.00E-36
53	hypothetical protein	-	27019	27657	636	212	100%	98.58%	[Staphylococcus phage Stau2]	9.00E-144
54	transcriptional regulator	-	27650	27865	213	71	100%	98.59%	[Staphylococcus phage vB_Sau-RP15]	7.00E-42
55	hypothetical protein	-	27879	28100	219	73	100%	98.63%	[Staphylococcus phage vB_SauM_Romulus]	2.00E-42
56	hypothetical protein	-	28202	28960	756	252	100%	98.02%	[Staphylococcus phage StAP1]	0
57	hypothetical protein	-	28974	29891	915	305	100%	99.34%	[Staphylococcus phage Koomba-kaat_1]	0
58	holin	-	29891	30391	498	166	100%	98.80%	[Staphylococcus phage Stau2]	3.00E-116
59	hypothetical protein	-	30472	30744	270	90	100%	98.89%	[Staphylococcus phage StAP1]	7.00E-58
60	transposase	-	30853	31452	597	199	100%	91.46%	[Staphylococcus phage Twort]	6.00E-128
61	tRNA	-	32053	32140	261	87	-	-	tRNA-Ser	-
62	ribosome associated inhibitor A	-	32925	33140	213	71	97%	79.71%	[Staphylococcus phage CF5]	3.00E-34

Table S1 List of annotated proteins from ORFs in Pelagios genome (cont.)

ORF	Predicted function	Direction	Start	Stop	Size (nt)	Size (aa)	Coverage	similarity	NCBI Sequence Hits	E-value
63	anti-repressor protein	-	33238	34101	861	287	63%	98.89%	[Staphylococcus phage PM56]	5E-124
64	hypothetical protein	-	34379	34594	213	71	100%	98.59%	[Staphylococcus phage SAPYZU_15]	8E-44
65	membrane protein	-	34605	34937	330	110	100%	70.91%	[Staphylococcus phage CF5]	2E-46
66	hypothetical protein	-	34944	35276	330	110	100%	98.18%	[Staphylococcus phage MR003]	3.00E-71
67	membrane protein	+	35720	36106	384	128	69%	98.86%	[Staphylococcus phage Koomba-kaat_1]	2.00E-55
68	hypothetical protein	+	36084	36365	279	93	100%	97.85%	[Staphylococcus phage vB_SauH_SPJ2]	3.00E-60
69	terminase small subunit	+	36362	36775	411	137	100%	76.64%	[Staphylococcus phage CF5]	2.00E72
70	terminase large subunit	+	36792	37160	336	112	95%	98.28%	[Staphylococcus phage CIFT_MFB_MRSA12]	3.00E-81
71	terminase large subunit	+	37422	37589	165	55	100%	100.00%	[Staphylococcus phage vB_SauM-HM01]	5.00E-32
72	MAG TPA: restriction enzyme	+	37830	37928	96	32	100%	90.62%	[Herelleviridae sp.]	5.00E-09
73	hypothetical protein	+	38136	39329	1191	397	94%	100.00%	[Staphylococcus phage Koomba-kaat_1]	0
74	hypothetical protein	+	39551	40471	918	306	82%	99.60%	[Staphylococcus phage Koomba-kaat_1]	0
75	Putative terminase large subunit	+	40548	40709	159	53	98%	96.15%	[Staphylococcus phage vB_SauM_VL10]	4.00E-27
76	hypothetical protein	+	40725	41525	798	266	100%	97.74%	[Staphylococcus phage SA11]	0
77	hypothetical protein	+	41678	42157	477	159	100%	93.71%	[Staphylococcus phage KSAP7]	9.00E-103
78	putative membrane-associated protein	+	42200	42949	747	249	100%	87.95%	[Staphylococcus phage vB_SauM_VL10]	5.00E-113
79	hypothetical protein	+	43010	43369	357	119	100%	99.16%	[Staphylococcus phage PBSA08]	1.00E-75
80	portal protein	+	43375	43740	363	121	100%	98.35%	[Staphylococcus phage Stau2]	1.00E-79
81	hypothetical protein	+	43745	44737	990	330	100%	99.09%	[Staphylococcus phage vB_SauM-HM01]	0
82	structure and packaging protein	+	45271	45975	702	234	100%	100.00%	[Staphylococcus phage PBSA08]	2E-169
83	hypothetical protein	+	46143	46916	771	257	100%	99.61%	[Staphylococcus phage SAC]	0
84	hypothetical protein	+	46930	47895	963	321	100%	97.82%	[Staphylococcus phage vB_SauM_Romulus]	0
85	hypothetical protein	+	47979	49370	1389	463	100%	95.03%	[Staphylococcus phage vB_SauM_VL10]	0
86	hypothetical protein	+	49474	49707	231	77	100%	89.41%	[Staphylococcus phage vB_SauM-V1SA19]	7.00E-41
87	hypothetical protein	+	49717	50466	747	249	100%	99.60%	[Staphylococcus phage phiRNIID]	0
88	hypothetical protein	+	50903	52111	1206	402	100%	99.75%	[Staphylococcus phage Stau2]	0
89	virion structural protein	+	52403	53278	873	291	100%	81.44%	[Staphylococcus phage CF5]	4.00E-174
90	hypothetical protein	+	53278	53898	618	206	100%	93.20%	[Staphylococcus phage SA11]	9.00E-135
91	MAG TPA: Baseplate hub assembly protein	+	53917	54753	834	278	99%	71.17%	[Herelleviridae sp.]	2.00E-143
92	hypothetical protein	+	54753	54959	204	68	100%	98.53%	[Staphylococcus phage vB_SauM-V1SA19]	6.00E-41
93	hypothetical protein	+	54982	56742	1758	586	100%	99.66%	[Staphylococcus phage vB_SauM-HM01]	0

Table S1 List of annotated proteins from ORFs in Pelagios genome (cont.)

ORF	Predicted function	Direction	Start	Stop	Size (nt)	Size (aa)	Coverage	similarity	NCBI Sequence Hits	E-value
94	tail tube protein	+	56805	57164	357	119	97%	93.04%	[Staphylococcus virus vB_SurM-PSU5]	3.00E-74
95	hypothetical protein	+	58070	59365	1293	431	100%	97.22%	[Staphylococcus phage OPT-SC01]	0
96	hypothetical protein	+	59382	59522	138	46	100%	91.30%	[Staphylococcus phage SAP24]	4.00E-19
97	hypothetical protein	+	59559	60008	447	149	100%	96.64%	[Staphylococcus phage phiRNIID]	2.00E-98
98	virion structural protein	+	60066	60374	306	102	100%	100.00%	[Staphylococcus phage SA11]	2.00E-66
99	tail assembly chaperone	+	60474	60890	414	138	99%	81.02%	[Staphylococcus phage CF5]	3.0E-79
100	tail morphogenetic protein	+	60982	61440	456	152	100%	67.10%	[Staphylococcus phage CF5]	4.00E-59
101	hypothetical protein	+	61494	64487	2991	997	100%	75.73%	[Staphylococcus phage vB_SauH_DELF3]	0
102	putative tail lysin	+	64930	65799	867	289	97%	79.21%	[Staphylococcus phage vB_SauH_DELF3]	2.00E-153
103	putative tail lysin	+	65858	66682	822	274	100%	73.36%	[Staphylococcus phage vB_SauH_DELF3]	1.00E-112
104	hypothetical protein	+	66734	69166	2430	810	100%	99.26%	[Staphylococcus phage KSAP11]	0
105	putative tail protein	+	69172	70065	891	297	84%	63.86%	[Staphylococcus phage SA5]	1.00E-115
106	hypothetical protein	+	70056	72512	2454	818	100%	98.78%	[Staphylococcus phage qdsa001]	0
107	hypothetical protein	+	72624	73475	849	283	100%	98.59%	[Staphylococcus phage Koomba-kaat_1]	0
108	virion structural protein	+	73475	73996	519	173	100%	78.03%	[Staphylococcus phage CF5]	1.00E-89
109	baseplate wedge subunit	+	73996	74700	702	234	100%	74.79%	[Staphylococcus phage CF5]	8.00E-129
110	hypothetical protein	+	74715	75761	1047	348	100%	99.14%	[Staphylococcus phage Koomba-kaat_1]	0
111	hypothetical protein	+	75772	78474	2700	900	100%	99.22%	[Staphylococcus phage vB_SauM-V1SA20]	0
112	virion structural protein	+	78578	79099	519	173	100%	99.42%	[Staphylococcus phage Stau2]	5.00E-122
113	hypothetical protein	+	79119	82577	3456	1152	100%	99.57%	[Staphylococcus phage MR003]	0
114	hypothetical protein	+	82629	82799	168	56	100%	100.00%	[Staphylococcus phage SA11]	1.00E-31
115	hypothetical protein	+	82789	84702	1911	637	100%	92.46%	[Staphylococcus phage SAC]	0
116	tail fiber protein	+	84718	85074	354	118	100%	86.44%	[Staphylococcus phage phiRNIID]	1.00E-67
117	hypothetical protein	+	85080	86444	1362	454	100%	86.78%	[Staphylococcus phage vB_SauM_VL10]	0
118	DNA helicase	+	86544	86999	453	151	100%	98.68%	[Staphylococcus phage Koomba-kaat_1]	6.00E-106
119	hypothetical protein	+	87221	88534	1311	437	100%	98.40%	[Staphylococcus phage vB_SauM-V1SA20]	0
120	hypothetical protein	+	88544	90130	1584	528	100%	97.35%	[Staphylococcus phage qdsa001]	0
121	hypothetical protein	+	90123	91562	1437	479	100%	99.79%	[Staphylococcus phage SapYZU04]	0
122	hypothetical protein	+	91562	91819	255	85	100%	98.82%	[Staphylococcus phage CIFT_MFB_MRSA12]	8.00E-56
123	putative holin	+	91889	92134	243	81	99%	62.50%	[Staphylococcus phage vB_SauH_DELF3]	2.00E-20
124	site-specific DNA methyltransferase	+	92144	92467	321	107	98%	81.90%	[Staphylococcus phage Quidividi]	4.00E-55

Table S1 List of annotated proteins from ORFs in Pelagios genome (cont.)

ORF	Predicted function	Direction	Start	Stop	Size (nt)	Size (aa)	Coverage	similarity	NCBI Sequence Hits	E-value
125	methyltransferase	+	92663	92863	198	66	100%	100.00%	[Staphylococcus phage SA11]	1.00E-39
126	hypothetical protein	+	92832	93080	246	82	100%	98.78%	[Staphylococcus phage Koomba-kaat_1]	7.00E-52
127	adenine-specific methyltransferase	+	93080	93769	687	229	100%	98.69%	[Staphylococcus phage vB_SauM-V1SA20]	4.00E-168
128	hypothetical protein	+	93839	94870	1029	343	100%	99.71%	[Staphylococcus phage vB_SauM-HM01]	0
129	hypothetical protein	+	94872	95138	264	88	100%	97.73%	[Staphylococcus phage qdsa001]	2.00E-56
130	hypothetical protein	+	95122	97065	1941	647	100%	98.45%	[Staphylococcus phage SAC]	0
131	anti-sigma factor	+	97049	97642	591	197	100%	99.49%	[Staphylococcus phage Koomba-kaat_1]	2.00E-138
132	hypothetical protein	+	97656	98732	1074	358	100%	98.32%	[Staphylococcus phage Koomba-kaat_1]	0
133	hypothetical protein	+	98793	99101	306	102	100%	99.02%	[Staphylococcus phage PM93]	3.00E-63
134	hypothetical protein	+	99101	99538	435	145	100%	90.67%	[Staphylococcus phage vB_SauM_VL10]	1.00E-88
135	RusA-like Holliday junction resolvase	+	99528	100136	606	202	99%	72.00%	HNH endonuclease [Staphylococcus phage phiRNIID]	2.00E-106
136	hypothetical protein	+	100151	101488	1335	445	100%	99.10%	[Staphylococcus phage vB_SauM_VL10]	0
137	hypothetical protein	+	101504	103711	2205	735	100%	99.73%	[Staphylococcus phage vB_Sau-RP15]	0
138	HNH endonuclease	+	103872	104588	718	238	100%	99.58%	[Staphylococcus phage phiRNIID]	8.00E-174
139	ribonucleotide reductase of class Ia	+	104802	105224	420	140	100%	100.00%	[Staphylococcus phage MR003]	4.00E-99
140	hypothetical protein	+	105237	105563	324	108	100%	97.22%	[Staphylococcus phage qdsa001]	5.00E-68
141	thioredoxin-like protein	+	105547	105867	318	106	100%	100.00%	[Staphylococcus phage Stau2]	1.00E-68
142	phage protein	+	106074	106676	600	200	100%	99.00%	[Staphylococcus phage MR003]	9.00E-141
143	putative integration host factor	+	106685	106996	309	103	100%	99.03%	[Staphylococcus phage CIFT_MFB_MRSA12]	7.00E-67
144	putative transposase	+	107282	107557	273	91	100%	97.80%	[Staphylococcus phage vB_SauM-V1SA20]	2.00E-58
145	hypothetical protein	+	107816	111565	3747	1249	100%	98.96%	[Staphylococcus phage vB_SauM-V1SA22]	0
146	HNH endonuclease	+	111808	112452	642	214	100%	99.53%	[Staphylococcus phage Stau2]	2.00E-156
147	putative DNA polymerase I	+	112676	113332	654	218	100%	98.62%	[Staphylococcus phage SAPYZU_15]	2.00E-154
148	MAG TPA: recombination, repair and ssDNA binding protein	+	113372	113854	480	160	100%	70.00%	[Herelleviridae sp.]	4.00E-78
149	hypothetical protein	+	113925	115085	1158	386	100%	95.14%	[Staphylococcus phage SA11]	0
150	hypothetical protein	+	115148	116383	1233	411	100%	98.78%	[Staphylococcus phage vB_Sau-RP15]	0
151	hypothetical protein	+	116376	116729	351	117	100%	99.15%	[Staphylococcus phage vB_SauM-V1SA22]	9.00E-79
152	sigma factor	+	116716	117375	657	219	100%	95.43%	[Staphylococcus phage qdsa001]	6.00E-151

ORF	Predicted function	Direction	Start	Stop	Size (nt)	Size (aa)	Coverage	similarity	NCBI Sequence Hits	E-value
153	Ig domain containing protein	+	117482	118114	630	210	100%	98.57%	[Staphylococcus phage SA11]	3.00E-148
154	major tail protein	+	118136	118648	510	170	100%	97.06%	[Staphylococcus phage CIFT_MFB_MRSA12]	7.00E-111
155	putative tail morphogenetic protein	+	118665	118877	210	70	100%	98.57%	[Staphylococcus phage CIFT_MFB_MRSA12]	2.00E-40
156	hypothetical protein	+	118974	119228	252	84	100%	98.81%	[Staphylococcus phage vB_SauM-V1SA22]	5.00E-49
157	hypothetical protein	+	119228	119998	768	256	100%	95.70%	[Staphylococcus phage StAP1]	3.00E-173
158	hypothetical protein	+	119991	121244	1251	417	100%	80.86%	[Staphylococcus phage vB_SauH_DELF3]	0
159	hypothetical protein	+	121256	121585	327	109	100%	98.17%	[Staphylococcus phage StAP1]	4.00E-67
160	hypothetical protein	+	121658	122191	531	177	100%	98.31%	[Staphylococcus phage vB_SauM-V1SA22]	1.00E-123
161	sce7726 family protein	+	122184	122945	759	253	97%	74.29%	[Staphylococcus phage vB_SscM-1]	3.00E-136
162	hypothetical protein	+	122926	123432	504	168	100%	98.21%	[Staphylococcus phage vB_SauM_Romulus]	3.00E-116
163	hypothetical protein	+	123432	124286	852	284	100%	98.59%	[Staphylococcus phage qdsa001]	0
164	hypothetical protein	+	124660	125391	729	243	100%	100.00%	[Staphylococcus phage SA11]	3.00E-174
165	virion structural protein	+	125409	125867	456	152	100%	92.76%	[Staphylococcus phage phiRNIID]	2.00E-97
166	hypothetical protein	+	125932	126369	435	145	100%	94.48%	[Staphylococcus phage KSAP7]	3.00E-91
167	hypothetical protein	+	126380	127015	633	211	100%	96.68%	Staphylococcus phage Koomba-kaat_1]	5.00E-146
168	hypothetical protein	+	127051	127449	396	132	100%	98.48%	[Staphylococcus phage vB_SauM-V1SA22]	1.00E-88
169	hypothetical protein	+	127590	127841	249	83	98%	95.06%	[Staphylococcus phage Koomba-kaat_1]	2.00E-46
170	hypothetical protein	+	127851	128114	261	87	100%	96.55%	[Staphylococcus phage Stau2]	3.00E-48
171	hypothetical protein	+	128115	128399	282	94	100%	96.81%	[Staphylococcus phage CIFT_MFB_MRSA12]	4.00E-59
172	hypothetical protein	+	128412	128705	291	97	100%	100.00%	[Staphylococcus phage Stau2]	1.00E-62
173	hypothetical protein	+	128769	129083	312	104	100%	97.12%	[Staphylococcus phage phiRNIID]	3.00E-69
174	hypothetical protein	+	129086	129331	243	81	100%	97.53%	[Staphylococcus phage SAC]	7.00E-47
175	hypothetical protein	+	129466	129960	492	164	100%	96.41%	[Staphylococcus phage vB_Sau-RP15]	9.00E-110
176	pentapeptide repeat family protein	+	129983	130579	594	198	100%	97.98%	[Staphylococcus phage St1.CM]	2.00E-136
177	hypothetical protein	+	130598	131149	549	183	100%	98.36%	[Staphylococcus phage SSP49]	3.00E-129
178	hypothetical protein	+	131155	131361	204	68	100%	98.53%	[Staphylococcus phage KSAP7]	1.00E-38
179	pentapeptide repeat-containing protein	+	131361	132080	717	239	100%	95.82%	[Staphylococcus phage vB_SauM-HM01]	8.00E-168
180	hypothetical protein	+	132067	132318	249	83	100%	98.80%	[Staphylococcus phage Stau2]	1.00E-46
181	hypothetical protein	+	132318	133217	897	299	100%	95.71%	[Staphylococcus phage CIFT_MFB_MRSA16]	0
182	hypothetical protein	+	133234	133758	522	174	100%	98.28%	[Staphylococcus phage MR003]	3.00E-117
183	hypothetical protein	+	133862	134059	195	65	100%	98.46%	[Staphylococcus phage qdsa001]	3.00E-37
184	hypothetical protein	+	134059	134454	393	131	100%	99.24%	[Staphylococcus phage BP13]	1.00E-88
185	pentapeptide repeat-containing protein	+	134451	134849	396	132	100%	60.12%	[Staphylococcus phage vB_SauM_Romulus]	1.00E-46
186	hypothetical protein	+	135003	135314	309	103	100%	99.03%	[Staphylococcus phage St1.CM]	3.00E-66
187	hypothetical protein	+	135440	135877	435	145	94%	91.67%	[Staphylococcus phage vB_SauM-V1SA20]	3.00E-45

A

	ORF1	ORF2	ORF3	ORF4	ORF5	ORF6	ORF7	ORF8	ORF9	ORF10	ORF11	ORF12	ORF13	ORF14	ORF15	ORF16	ORF17	ORF18	ORF19	ORF20	
CSM-toxin	0.8	0	0	0	0.06	0.01	0	0	0	0	0.47	0.02	0	0	0.19	0	0	0	0	0	Probability
CSM-toxin	+	-	-	-	-	-	-	-	-	-	-	-	-	-	-	-	-	-	-	-	Prediction
ToxinPred3.0	0	0	0	0	0	0	0	0	0	0	0	0.12	0	0	0	0	0	1	0	0	Probability
ToxinPred3.0	-	-	-	-	-	-	-	-	-	-	-	-	-	-	-	-	-	+	-	-	Prediction

Server	ORF21	ORF22	ORF23	ORF24	ORF25	ORF26	ORF27	ORF28	ORF29	ORF30	ORF31	ORF32	ORF33	ORF34	ORF35	ORF36	ORF37	ORF38	ORF39	ORF40	
CSM-toxin	1	0.07	0	0	0	0	0	0	0.69	0.88	0.01	0.73	0	0	0.01	0	0.01	0	0	0	Probability
CSM-toxin	-	-	-	-	-	-	-	-	+	-	-	-	-	-	-	-	-	-	-	-	Prediction
ToxinPred3.0	0	0	0	0	0.11	0	0	0	0	0	0	0	0	0	0	0	0	0	0	0	Probability
ToxinPred3.0	-	-	-	-	-	-	-	-	-	-	-	-	-	-	-	-	-	-	-	-	Prediction

Server	ORF41	ORF42	ORF43	ORF44	ORF45	ORF46	ORF47	ORF48	ORF49	ORF50	ORF51	ORF52	ORF53	ORF54	ORF55	ORF56	ORF57	ORF58	ORF59	ORF60	
CSM-toxin	0	0	0	0	0	0	0	0	0	0	0	0	0	0	0	0	0.07	0	0	0	Probability
CSM-toxin	-	-	-	-	-	-	-	-	-	-	-	-	-	-	-	-	-	-	-	-	Prediction
ToxinPred3.0	0	0	0	0	0	0	0	0	0	0	0	0	0	0	0	0	0	0	0	0	Probability
ToxinPred3.0	-	-	-	-	-	-	-	-	-	-	-	-	-	-	-	-	-	-	-	-	Prediction

Server	ORF61	ORF62	ORF63	ORF64	ORF65	ORF66	ORF67	ORF68	ORF69	ORF70	ORF71	ORF72	ORF73	ORF74	ORF75	ORF76	ORF77	ORF78	ORF79	ORF80	
CSM-toxin	0	0	0	0	0	0	0	0	0	0	0	0.01	0	0	0	0	0	0	0	0.96	Probability
CSM-toxin	-	-	-	-	-	-	-	-	-	-	-	-	-	-	-	-	-	-	-	-	Prediction
ToxinPred3.0	0	0	0	0	0	0	0.07	0	0	0	0	0	0	0	0	0	0	0	0	0.15	Probability
ToxinPred3.0	-	-	-	-	-	-	-	-	-	-	-	-	-	-	-	-	-	-	-	-	Prediction

Server	ORF81	ORF82	ORF83	ORF84	ORF85	ORF86	ORF87	ORF88	ORF89	ORF90	ORF91	ORF92	ORF93	ORF94	ORF95	ORF96	ORF97	ORF98	ORF99	ORF100	
CSM-toxin	0	0.03	0	0	0	0	0	0	0	0	0	0.11	0	0	0	0.01	0.01	0	0.8	0.79	Probability
CSM-toxin	-	-	-	-	-	-	-	-	-	-	-	-	-	-	-	-	-	-	-	-	Prediction
ToxinPred3.0	0	0	0	0	0	0	0.31	0	0	0	0	0	0	0	0	0	0	0	0.18	0	Probability
ToxinPred3.0	-	-	-	-	-	-	-	-	-	-	-	-	-	-	-	-	-	-	-	-	Prediction

Server	ORF101	ORF102	ORF103	ORF104	ORF105	ORF106	ORF107	ORF108	ORF109	ORF110	ORF111	ORF112	ORF113	ORF114	ORF115	ORF116	ORF117	ORF118	ORF119	ORF120	
CSM-toxin	0	0.97	0	0.08	0	0	0	0	0.99	0	1	0	1	0	1	0.37	0	0.06	0	0	Probability
CSM-toxin	-	-	-	-	-	-	-	-	-	-	-	-	-	-	-	-	-	-	-	-	Prediction
ToxinPred3.0	0	0	0	0	0	0	0	0	0	0	0	0	0	0	0	0	0	0	0.08	0	Probability
ToxinPred3.0	-	-	-	-	-	-	-	-	-	-	-	-	-	-	-	-	-	-	-	-	Prediction

Server	ORF121	ORF122	ORF123	ORF124	ORF125	ORF126	ORF127	ORF128	ORF129	ORF130	ORF131	ORF132	ORF133	ORF134	ORF135	ORF136	ORF137	ORF138	ORF139	ORF140	
CSM-toxin	0.22	0	0	0	0	0	0	0	0	0.12	0.15	0	0	0	0.07	0	0	0	0	0	Probability
CSM-toxin	-	-	-	-	-	-	-	-	-	-	-	-	-	-	-	-	-	-	-	-	Prediction
ToxinPred3.0	0	0	0	0.20	0	0	0	0	0	0	0	0	0	0	0	0	0	0	0	0.36	Probability
ToxinPred3.0	-	-	-	-	-	-	-	-	-	-	-	-	-	-	-	-	-	-	-	-	Prediction

Server	ORF141	ORF142	ORF143	ORF144	ORF145	ORF146	ORF147	ORF148	ORF149	ORF150	ORF151	ORF152	ORF153	ORF154	ORF155	ORF156	ORF157	ORF158	ORF159	ORF160	
CSM-toxin	0.83	0	0	0.16	0	0	0.2	0	0	0	0.02	0	0	0	0	0	0	0	0	0	Probability
	-	-	-	-	-	-	-	-	-	-	-	-	-	-	-	-	-	-	-	-	Prediction
ToxinPred3.0	0	0	0	0	0	0	0	0	0	0	0	0	0	0	0	0.22	0	0	0	0	Probability
	-	-	-	-	-	-	-	-	-	-	-	-	-	-	-	-	-	-	-	-	Prediction

Server	ORF161	ORF162	ORF163	ORF164	ORF165	ORF166	ORF167	ORF168	ORF169	ORF170	ORF171	ORF172	ORF173	ORF174	ORF175	ORF176	ORF177	ORF178	ORF179	ORF180	
CSM-toxin	0	0	0	0	0	0	0	0	0	0	0	0.48	0	0	0	0	0	0	0.01	0	Probability
	-	-	-	-	-	-	-	-	-	-	-	-	-	-	-	-	-	-	-	-	Prediction
ToxinPred3.0	0	0	0	0	0	0.24	0	0	0	0	0	0	0	0	0	0	0	0	0	0	Probability
	-	-	-	-	-	-	-	-	-	-	-	-	-	-	-	-	-	-	-	-	Prediction

Server	ORF181	ORF182	
CSM-toxin	0	0	Probability
	-	-	Prediction
ToxinPred3.0	0	0	Probability
	-	-	Prediction

	Search	Prediction
DBETH server	Toxin sequences	low identity, very low query coverage and non-significant E-value<br>The sequence is not strongly predicted to be a toxin.
	Toxin domains from<br>- CDD database<br>- SMART database	 No hit found<br>No hit found

B

	Web-server	Prediction
**Virulence factor (VF) prediction**	VFanalyzer (VFDB)	No VF found
	VirulenceFinder 2.0<br>-organism: E. coli<br>-organism: S. aureus	 No hit found<br>No hit found

Figure S1. **(A)** Toxin proteins predicted from Pelagios genome using CSM-toxin, ToxinPred3.0, and DBETH web servers. CSM-toxin and ToxinPred3.0 show the probability for ORFs as a toxin in the upper panel and indicate the prediction result in the lower panel (+; toxin or -; non-toxin). **(B)** Virulence factor prediction using VFanalyzer from the virulence factor database (VFDB) and VirulenceFinder 2.0 web servers. The absence of toxin genes and virulence factors in the genome of Phipelagios suggests that the phage is safe and has a low risk of transferring toxins or virulence factor genes to its bacterial host

Antimicrobial class: aminoglycoside; aminocyclitol

Antimicrobial	gentamicin	tobramycin	streptomycin	amikacin	isepamicin	dibekacin	kanamycin	neomycin	lividomycin	paromomycin	ribostamycin	unknown aminoglycoside	butiromycin	butirosin	hygromycin	netilmicin	apramycin	sisomicin	arbekacin	kasugamycin	astromicin	fortimicin	spectinomycin
Resistance predicted	-	-	-	-	-	-	-	-	-	-	-	-	-	-	-	-	-	-	-	-	-	-	-

Antimicrobial class: quinolone; beta-lactam

Antimicrobial	fluoroquinolone	ciprofloxacin	unknown quinolone	nalidixic acid	amoxicillin	amoxicillin+clavulanic acid	ampicillin	ampicillin+clavulanic acid	cefepime	cefixime	cefotaxime	cefoxitin	ceftazidime	ertapenem	imipenem	meropenem	piperacillin	piperacillin+tazobactam	unknown beta-lactam	aztreonam	cefotaxime+clavulanic acid	temocillin	ceftazidime+avibactam	penicillin	ceftriaxone
Resistance predicted	-	-	-	-	-	-	-	-	-	-	-	-	-	-	-	-	-	-	-	-	-	-	-	-	-

Antimicrobial class: beta-lactam; under_development; folate pathway antagonist; fosfomycin; glycopeptide; lincosamide; streptogramin a; pleuromutilin; macrolide

Antimicrobial	ticarcillin+clavulanic acid	cephalothin	piperacillin+clavulanic acid	ceftiofur	sulfamethoxazole	trimethoprim	fosfomycin	vancomycin	teicoplanin	bleomycin	lincomycin	clindamycin	dalfopristin	pristinamycin iia	virginiamycin m	quinupristin+dalfopristin	tiamulin	carbomycin	erythromycin	azithromycin	oleandomycin	spiramycin	telithromycin	telithromycin
Resistance predicted	-	-	-	-	-	-	-	-	-	-	-	-	-	-	-	-	-	-	-	-	-	-	-	-

Antimicrobial class: tetracycline; streptogramin b; oxazolidinone; amphenicol; polymyxin; steroid antibacterial; pseudomonic acid; rifamycin; nitroimidazole; ionophores

Antimicrobial	tetracycline	doxycycline	minocycline	tigecycline	quinupristin	pristinamycin ia	virginiamycin s	linezolid	chloramphenicol	florfenicol	colistin	fusidic acid	mupirocin	rifampicin	metronidazole	narasin	salinomycin	maduramicin
Resistance predicted	-	-	-	-	-	-	-	-	-	-	-	-	-	-	-	-	-	-

Figure S2. Prediction of antimicrobial resistance genes of the Pelagios genome using ResFinder version 4.7.2. The hyphen (-) indicates no resistance and no match found in the database. The results

showed that no antibiotic resistance genes were detected in Pelagios, suggesting that it is safe and unlikely to transfer resistance genes to its bacterial host.
